# Topology-optimized melt-electrowritten PCL patch for abdominal wall reconstruction

**DOI:** 10.1016/j.bioactmat.2025.09.026

**Published:** 2025-10-01

**Authors:** Yakui Liu, Max von Witzleben, Sarah Duin, Anne Bernhardt, Michael Gelinsky

**Affiliations:** Centre for Translational Bone, Joint and Soft Tissue Research, University Hospital Carl Gustav Carus and Faculty of Medicine, Technische Universität Dresden, Fetscherstr. 74, 01307, Dresden, Germany

**Keywords:** Melt electrowriting, Abdominal patch, Topology optimization, Regenerative medicine

## Abstract

Abdominal wall patches are clinically essential for treating abdominal defects or hernias, with mechanical strength representing a critical requirement. Therefore, rational scaffold design and fabrication methods are crucial for achieving optimal performance. This study introduces an innovative approach to the design of scaffolds for abdominal wall repair, using topology optimization and melt electrowriting (MEW). Through topology optimization, we provided a systematic, data-driven basis for scaffold design. We further refined the scaffold structure to enhance print efficiency and continuity, and successfully implemented MEW as fabrication technology, marking its first application in abdominal repair. Mechanical testing revealed that the topology-optimized scaffold achieved abdominal tensile strength of 1.85 ± 0.02 N/cm, 39 % superior to conventional designs. Subsequent biological assessments – including fibroblast proliferation and alignment analyses – showed that collagen coating significantly enhanced cell attachment and proliferation, especially in multi-layer (300 layers) scaffolds, maintaining diameters of 11.34 ± 0.67 μm throughout the depth. Finally, *ex vivo* porcine abdominal wall tests confirmed clinical mechanical suitability. This work offers a promising direction for future advancements in tissue engineering, particularly in optimizing scaffold structures for biological and mechanical performance.

## Highlights

• The topologically optimized scaffold with a tessellated octagonal grid-structure, successfully fabricated via MEW, achieves the required mechanical properties for abdominal wall repair.
• Collagen coating enhances cell adhesion, proliferation, and deep penetration, particularly in thick multilayer (300-layer) scaffolds.
• Biomechanical and *ex vivo* validation confirm that the scaffold surpasses the clinical tensile strength requirement (16 N/cm).

## Graphical abstract

**Figure:**
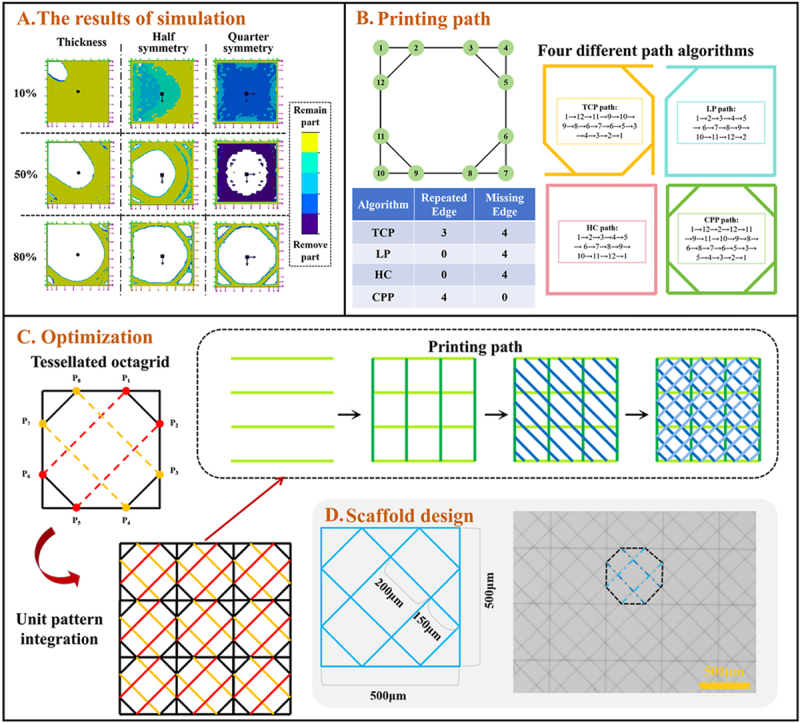

## 1 Introduction

The human abdominal wall is composed primarily of skin, adipose tissue, muscle, preperitoneal fascia, and peritoneum, which are interconnected to provide sufficient mechanical strength and elasticity to contain the organs in the abdominal cavity [1]. An abdominal hernia occurs when a weak, defective, and injured area of the abdominal wall results in an abdominal organ or part of any organ protruding [2]. Clinically, surgeons usually use sutures or surgical patches to treat abdominal hernias [3]. To achieve effective abdominal wall repair, patches must possess multiple essential properties, including adequate mechanical strength, antimicrobial activity, and anti-adhesive characteristics. Among these, mechanical properties are the most critical, as the patch must exhibit a tensile strength of at least 16 N/cm [4] to provide sufficient support for daily physical activities.

Currently, the polypropylene (PP) patch is the most widely used commercial patch. Its effectiveness in hernia repair was first demonstrated in the 1950s by the American surgeon Usher in an animal model [5]. In recent years, new hernia repair patches have emerged because of the rapid development of health care, advances in medical science and technology, and improvements in materials research. Since natural materials usually do not provide sufficient mechanical properties, most of the materials currently used for patches in both clinic and research are synthetic [6]. Based on this, the current techniques for the preparation of patches are mainly knitting and weaving, 3D printing, and electrospinning [7,8].

Conventional textile weaving and 3D printing technologies (mainly fused filament fabrication, FFF) offer millimeter-scale control of pore geometry. However, the resulting filaments are too thick and hydrophobic to guide cellular organization [9,10]. Electrospinning, by contrast, can generate fibrous filaments with diameters in the nanometer scale to prepare sheet-like scaffolds with larger surface areas. Moreover, nanoscale fibers can closer mimic the extracellular matrix, thus facilitating cell adhesion and growth. However, due to smaller fiber filaments, electrospun patches are often not able to provide sufficient mechanical support. In addition, solution-based electrospinning typically involves potentially cytotoxic solvents that can adversely affect cell growth, and it does not allow precise control over fiber deposition, thus limiting the specific design of the patch structure [11].

In recent years, in the field of additive manufacturing a novel technique evolved, melt electrowriting (MEW), capable of accurately depositing continuous polymer microfibers to create high-resolution structures [12]. MEW can be seen as a technology that combines 3D printing and electrospinning, which can precisely control the diameter as well as the distribution of the fiber filaments and is able to create scaffolds with complex geometries. At the same time, it has fiber filaments in the micro-meter scale, which support cell adhesion and oriented cell growth [13]. To date, MEW has proven to be a promising enabling tool in regenerative medicine, offering great potential for the design of tissue-specific ECM-like scaffolds, healthy as well as pathophysiological microenvironments, and personalized and potentially functional implants [[14], [15], [16]]. The gold standard polymer currently used for MEW is polycaprolactone (PCL), a semi-crystalline, biodegradable polymer [17]. Due to its low melting point and rapid solidification, it is easy to process through MEW. Medical-grade PCL has excellent biocompatibility, a suitable degradation rate, and good mechanical properties; therefore, it has a broad application prospect in tissue engineering [18,19]. In summary, MEW has great potential for preparing abdominal wall patches that meet the clinical needs: on the one hand, the diameter of PCL filaments and the structure of the patch can be adjusted to tailor its mechanical requirements; on the other hand, the microfibers are able to further guide the growth of the cells and accelerate the repair of the tissues. At this point, there is only one critical question left: which structure is better suited for the abdominal wall patch?

At present, the structural design of abdominal wall patches still remains in the stage of trial and error, mostly using simple geometrical shapes, such as squares or rhombuses [6], and does not provide a strong theoretical support for the design of the structure. More and more studies focus on how to rationally design scaffold structures to achieve better repair of specific tissues [20,21]. Parametric design—a broad category in which geometry is generated by systematically varying underlying parameters—has emerged as a powerful framework for implant design. Optimization of structures using parametric design (based on algorithms) is an approach with high potential for the structural design of implants. One of the most effective and widely used methods is topology optimization which is a method to optimize the material distribution in a given region based on given load conditions, constraints and performance indicators [22]. The application of topology optimization enhances the efficiency and precision of scaffold and implant design, enabling them to better adapt to the complex conditions in biological environments [23,24]. This approach not only optimizes the mechanical properties of structures, but also promotes the growth and regeneration of new tissues, making it an important technical tool in the field of tissue engineering and regenerative medicine [25].

Given these limitations, this study aims to develop an advanced abdominal wall scaffold by leveraging the precision and versatility of MEW combined with a topology optimization approach. By integrating MEW, we sought to overcome the constraints of traditional fabrication methods, enabling the production of scaffolds with tailored mechanical properties, optimized geometry, and fine fiber structures that promote cell growth, making them particularly suitable for abdominal wall repair. The application of topology optimization ensures a rational, data-driven design process, moving beyond trial-and-error approaches to create scaffolds with enhanced stress distribution and mechanical performance. Ultimately, our work aims to provide a clinically relevant solution that meets both the mechanical and biological demands for effective abdominal wall repair.

## 2 Material and methods

### 2.1 Topology simulation

In this study, Topology optimization simulations were carried out using SolidWorks Simulation 2023's Topology Study module (Dassault Systèmes, France), which utilizes a Solid Isotropic Material with Penalization (SIMP) algorithm [26]. The objective of the topology study was to minimize the part's mass while keeping satisfying constraints. The mechanical strength of the patch, benchmarked against the gold standard of 16 N/cm [4], was identified as the critical performance metric for evaluation. Consequently, the simulation model and boundary conditions were meticulously crafted to ensure alignment with this mechanical criterion.

The simulation model was constructed as a solid rectangular structure, with precise dimensions of 10 mm × 10 mm × 0.1 mm (Fig. S1A), representing a simplified abstraction of the patch's material. Boundary conditions were applied such that the upper and left edges of the model were fixed, while a tensile force of exactly 16 N was uniformly distributed across the right and lower edges (Fig. S1B), simulating the forces experienced in practical applications. To enhance the robustness of the simulation, control conditions including half symmetry, quarter symmetry, and thickness were implemented (Fig. S1C), ensuring a comprehensive evaluation of the structure's performance under varied conditions.

### 2.2 Fabrication and treatment of MEW scaffolds

The MEW printing procedures were performed using a GeSiM BioScaffolder 3.1 system, integrated with a specialized MEW print head (GeSiM, Radeberg, Germany). Medical-grade polycaprolactone (PCL; Purasorb PC 12, Corbion, Amsterdam, Netherlands) was employed as the base material. The PCL was heated to 79 °C to ensure optimal melt flow properties, after which it was extruded into fine fibers under an applied electric field of 7 kV. A nozzle-to-collector distance of 3.5 mm was maintained as part of the controlled conditions. The printing process was carried out at a speed of 700 mm/min with an applied pressure of 20 kPa, achieving a balance between fiber precision and deposition stability. These parameters were selected based on extensive experimental experience in our group [14] and established MEW studies using PCL, which consistently report similar processing windows for stable jet formation and reproducible fiber morphology [17,27].

To further enhance the bioactivity of the MEW-fabricated scaffolds, a collagen coating was applied to the surface. Prior to this, the scaffolds underwent a hydrophilization process to increase surface hydrophilicity, a crucial step for improving the adhesion and uniformity of the coating. The scaffolds were immersed in a 5 M sodium hydroxide (NaOH) solution for 1 h to chemically modify the surface. After this treatment, they were thoroughly rinsed with deionized water to remove residual NaOH, followed by air drying at room temperature.

Collagen type I, derived from rat tail (Meidrix Biomedicals, Esslingen, Germany), was selected for coating due to its excellent biocompatibility and its potential to promote cellular attachment. Similar to previous work [14,15], the scaffolds were submerged in a collagen solution (1 mg/mL in 0.1 M acetic acid), followed by neutralizing with 1 M NaOH. This mixture was incubated at 37 °C for 30 min to ensure both fibrillization and adsorption of collagen onto the scaffold surfaces. Following incubation, excess liquid was removed by gently placing the scaffold on top of filter paper. The scaffolds were then allowed to air-dry at room temperature, resulting in a uniformly collagen-coated MEW scaffold.

### 2.3 Mechanical tests

Tensile tests were conducted using a universal testing machine (Z010 equipped with a 100 N load cell, ZwickRoell, Germany) to evaluate the mechanical properties of the scaffolds. Custom-designed fixtures, specifically fabricated using a stereolithography (SLA) printer (Formlabs 3, Formlabs, Germany), were employed to ensure precise alignment and secure gripping of the scaffold during testing. The testing speed was consistently maintained at 1 mm/min to provide a controlled strain rate, allowing for accurate measurement of the scaffold's mechanical response. Each scaffold specimen was subjected to uniaxial tension until complete structural failure, with data recorded throughout the process to assess key mechanical parameters.

In clinical practice for abdominal wall repair, the tensile strength of scaffolds is often expressed in N/cm rather than the more conventional stress units (e.g., Pa). This is because abdominal wall patches are typically quite thin, allowing the thickness to be treated as negligible [4]. In this paper, all subsequent mechanical strengths of scaffolds will be expressed as abdominal tensile strength (N/cm).

### 2.4 *In vitro* cytocompatibility characterization of MEW scaffolds

#### 2.4.1 Cell culture and seeding on MEW scaffolds

Normal Human Dermal Fibroblasts (NHDF) were obtained from Promocell (Heidelberg, Germany) and cultured to passages 10–12 in Dulbecco's Modified Eagle's Medium (DMEM), supplemented with 10 % fetal calf serum (FCS, Gibco), 100 U/mL penicillin, and 100 μg/mL streptomycin (P/S). The culture medium was refreshed every three days.

For cell inoculation, 24- or 12-well plates (Corning Costar, New York, USA) were utilized depending on experimental requirements. All scaffolds were disinfected by immersion in 70 % ethanol for 20 min, followed by a 30 min rinse in phosphate-buffered saline (PBS) to remove residual ethanol. After disinfection, the scaffolds were transferred into the well plates and immersed in 1 mL of serum-free medium for 20 min to prepare them for cell seeding. Subsequently, the medium was aspirated, and a drop of NHDF cell suspension was carefully placed on top of each scaffold, ensuring uniform cell distribution. Cell numbers for seeding of different scaffold geometries are listed in Table 1.

**Table 1:** Experimental plan-cell culture experiments.

Scaffold size	Cell number	Method
10 mm × 10 mm × 4 layers	1 × 10^5^	Live/dead & DAPI/phalloidin/vimentin
5 mm × 5 mm × 4 layers	2 × 10^4^	LDH &DNA
10 mm × 10 mm × 40 layers	8 × 10^4^	Live/dead & DAPI/phalloidin & biomechanics
10 mm × 10 mm × 300 layers	2 × 10^5^	Live/dead & DAPI/phalloidin/vimentin

The scaffolds, now seeded with cells, were incubated for 2 h at 37 °C to promote initial cell attachment. After the incubation period, 1 mL of fresh, complete cell culture medium was gently added to each sample. The medium was replaced every three days to maintain a suitable environment for cell growth throughout the experiment.

#### 2.4.2 Cell biological analyses

***Live/dead staining:*** Cell viability was assessed by a live and dead staining using Calcein AM and Ethidium homodimer-1 (EthD-1) (Molecular Probes Inc.) at day 1, 7, 14, 21, and 28. Calcein stained the metabolically active cells in green and EthD-1 the dead cells in red. Imaging was performed with a fluorescence microscope (Keyence BZ 9000, Osaka, Japan).

***Fluorescent immunocytochemistry staining:*** After washing with PBS, cells were fixed with 4 % formaldehyde in PBS (SAV LP, Flintsbach a. Inn, Germany) for 45 min. Then, the scaffolds were washed twice with PBS and permeabilized for 5 min using 0.1 % Triton X-100 (Serva, Heidelberg, Germany). After that, the scaffolds were washed twice with PBS and were blocked with 3 % bovine serum albumin (BSA) (Carl Roth, Karlsruhe, Germany) in PBS for 1 h. Subsequently, the scaffolds were washed twice with 0.1 % Tween-20 in PBS and then incubated with primary antibody against vimentin (ABIN6284417, host – rabbit, 1:1500 in 1 % BSA) at 4 °C overnight. Then, scaffolds were washed twice with 0.1 % Tween-20 in PBS. The secondary antibody (AlexaFluor 546 goat anti-rabbit, 1:500, Thermo Fisher Scientific) was coupled with Phalloidin iFLuor 488 (1:1000, Abcam, Cambridge, UK) to stain cytoskeletal F-actin filaments and 4′,6-diamidino-2-phenylindole (DAPI) (1 μg/mL) (AppliChem, Darmstadt, Germany) to stain the nuclei, diluted in 1 % BSA. Incubation of the scaffolds was carried out at 4 °C overnight, protected from light. Imaging was conducted with a fluorescence microscope (Keyence BZ 9000, Osaka, Japan), while a confocal microscope (Leica Microsystems, Wetzlar, Germany) was used for multilayer scaffolds. To assess cell infiltration along the Z-axis, all confocal DAPI Z-stack images were compiled, and the “Plot Z-axis Profile” function in ImageJ was used to quantify fluorescence intensities.

***Quantification of cell number:*** With established lab protocols DNA and kinetic lactate dehydrogenase (LDH) assays were performed to quantify cell numbers at different time points (d1, d7, d14, d28, d35) [14]. At each time point, the scaffolds were washed with PBS and frozen at −80 °C until examination. After defrosting the scaffolds, cell lysates were obtained by immersing the scaffolds in 1 % Triton-X 100 for 50 min (including a sonication bath on ice for 10 min). For the DNA assay, the measurement was carried out by using a Quantifluor dsDNA kit (Promega, Madison, WI, USA) according to the manufacturer's instructions. The required 10 μl of the cell lysates were transferred in triplicate into a black flat 96-well plate, and 190 μl of the Quantifluor coloring solution was added to each sample. The plate was then protected from light and incubated at room temperature for 5 min. Fluorescence at 485 nm ex/535 nm em was measured using a multifunction microplate reader (InfinitePro, Tecan, Männedorf, Switzerland), and relative fluorescence values were correlated with cell numbers using a cell calibration line. The activity of intracellular LDH was quantified in the same lysates using the CytoTox 96® Non-Radioactive Cytotoxicity Assay (Promega, Madison, WI, USA). The required 10 μl of the cell lysates were transferred in triplicate into a transparent 96-well plate. Then, 40 μl lysis buffer and 50 μl substrate solution were added to each sample in sequence. Finally, the absorbance at 490 nm was recorded for 5 min. The slopes of the absorbance were correlated with the slopes measured with identically treated lysates of known cell numbers, which acted as a calibration curve. The measured intensity was correlated with the cell numbers by using a calibration line.

***Orientation analysis:*** The analysis of cell orientation was mainly carried out using Image J. First, 10x images of DAPI, Vimentin, and Phalloidin from single channel from D1, D7, and D14 samples were converted to 8-bit images and the brightness and contrast of the images were adjusted. Subsequently, the images were converted to binary ones by threshold control. The orientation was evaluated using the ImageJ plug-in OrientationJ provided by the Biomedical Imaging Group of EPFL, Switzerland (download: http://bigwww.epfl.ch/demo/orientation/). Specifically, the feature “OrientationJ Distribution” was executed with an object size of one pixel and the cubic spline gradient with the parameters of 60 % coherence and 1 % energy. Three sets of fluorescent photographs from different locations were selected for each set of scaffolds to be used for measurements, and the measured data were finally visualized using Origin 2019.

#### 2.4.3 Setup of different cell experiments

In order to be able to evaluate the biological performance of the scaffolds more efficiently, different sizes of scaffolds were designed for different cellular experiments. The specific schemes are shown in Table 1. All cell experiments contained two groups (experimental group: collagen-coated scaffolds; control group: pure PCL scaffold)

### 2.5 Scanning electron microscopy (SEM) analysis

The microstructures of the scaffolds, both with and without cells, were examined using scanning electron microscopy (SEM). For cell-seeded scaffolds, the samples were first fixed with 4 % formaldehyde (FA) solution at room temperature. Following fixation, the samples underwent a graded ethanol dehydration series: 10 %, 30 %, 50 %, 70 %, 80 %, 96 %, and twice in 99.9 % ethanol, each step lasting 10 min with gentle shaking. After dehydration, excess ethanol was carefully removed using filter paper, and the samples were left to dry in a fume hood at room temperature. The dried cell-laden scaffolds and the acellular scaffolds were then mounted onto SEM stubs, sputter-coated with a thin layer of gold to enhance conductivity, and imaged using a Zeiss DSM 982 Gemini scanning electron microscope (Carl Zeiss, Oberkochen, Germany).

### 2.6 *Ex vivo* application model

Fresh abdominal wall tissues from cadaver pigs ( provided by Pulmonary Engineering Group, Department of Anesthesiology and Intensive Care Medicine, University Hospital Carl Gustav Carus) were collected and frozen within 2 h post-harvest. The secondary use of this cadaveric porcine tissue for abdominal wall patch testing did not require additional ethical approval, as confirmed by the institutional Animal Ethics Committee, since the tissue originated from a pre-approved animal study. Then uniformly thawed on the day of the experiment. The tissue samples, comprising both skin and muscle layers, were cut into approximately 10 cm × 10 cm sections for subsequent wound repair simulations.

A 1 cm × 1 cm square defect was created at the center of each tissue sample using a scalpel to simulate an abdominal wall defect. Care was taken to ensure that the edges of the defect were smooth. Based on the experimental design, the repair patch was trimmed to match the defect size (approximately 1 cm × 1 cm) and secured over the defect using standard surgical suturing techniques. Absorbable sutures (V1225, Ethicon, Johnson & Johnson MedTech, USA) were used, with three stitches placed on each side of the patch. The suturing ensured that the patch firmly adhered to the tissue without any loosening or displacement.

To evaluate the mechanical integrity of the repaired tissue, all samples underwent tensile testing as described in section 2.3.

### 2.7 Statistical analysis

All experimental results were expressed as mean ± standard deviation and analyzed using one-way ANOVA followed by Tukey's test for multiple comparisons in the GraphPad Prism 10 software. The level of significance is noted by ∗, ∗∗, and ∗∗∗, which refer to the P values of <0.05, <0.01, and <0.001, respectively.

## 3 Results and discussion

### 3.1 Topology simulation

**Fig. 1:**
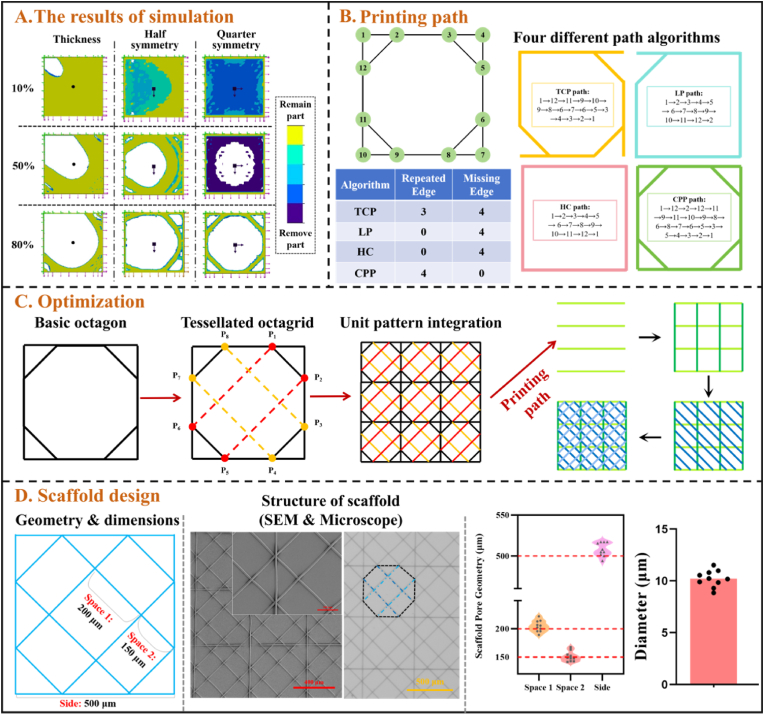
**Topology optimization, path planning, and scaffold design for abdominal wall patch**. (A) Simulation Results: Topology optimization outcomes at 10 %, 50 %, and 80 % material retention, considering different control conditions. (B) Path Planning: Comparison of four path algorithms—TCP, LP, HC, CPP—for efficient scaffold printing, highlighting repeated and missing edges for each method. (C) Optimized Scaffold Design: A tessellated octagonal grid was developed, and the printing path was optimized for layer-by-layer construction. (D) Geometry and characterization of the fabricated scaffolds. *Left*: scaffold aperture design and dimensions; *Middle*: representative SEM micrographs at two magnifications and a brightfield optical image confirm reproduction of the designed geometry; *Right*: Quantitative analysis of pore geometry and fiber diameter, red dashed lines denote design values (n = 10).

To obtain optimized patch structures, we compared the results under thickness control, half symmetry, and quarter symmetry control conditions and compared the structural evolution under each condition for different weight reduction percentages. From a MEW perspective, it is important to note that the 0 % weight reduction model represents a fully solid block, rather than a conventional MEW scaffold. We include it here primarily as a baseline to illustrate how the structure evolves from a dense, unoptimized form into a progressively refined design suitable for MEW fabrication. Such a scaffold is purely theoretical as a dense fiber layer cannot be printed due to the inherent electrostatic forces of the fibers [28]. In order to visualize the structural evolution during the optimization process, we summarize and compare the optimization results for each control condition at 10 %, 50 % and 80 % weight reduction percentages (Fig. 1A). The complete experimental results can be seen in Figs. S2–S4.

Under the thickness control condition, as the weight is reduced, the optimization results showed a gradual decrease in the retained material area. Especially at 80 % weight reduction, the retained structure has been significantly simplified and is mainly concentrated in the edge frame area. This structural optimization minimizes non-essential materials, which significantly reduces the weight of the patch, especially at high percentages (70 %–90 %) where the optimized design becomes extremely streamlined. However, the retained areas progressively decrease, especially under 90 % reduction (Fig. S2), leading to highly intricate and fragmented structures that significantly increase the difficulty of fabrication.

When the control condition is half symmetry, the region of retained material remains relatively large and maintains good symmetry over the course of 10–40 % weight reduction (Fig. S3). At 50–80 %, the structure becomes more regular, and the retained material shows a symmetrical distribution that gradually shrinks to the center and edge regions. Especially at 70–80 % weight reduction, the retained material forms a frame-like structure that distributes the stresses more uniformly, in line with the need for symmetric load distribution in biomedical applications. At 90 %, the system may not be able to compute an effective structure and may even collapse due to excessive material removal. This means that at 90 % weight reduction, the design may be close to its limits and unable to maintain basic structural integrity. In conjunction with the previous discussion, a 70–80 % optimized structure may be more appropriate for the MEW process.

Although semi-symmetric structures have provided a stable and feasible optimization scheme, the complexity of the symmetry may directly affect the distribution of mechanical properties of the patch in practical applications. This is because, in abdominal wall defect repair, the patch needs to withstand biological stresses from all directions. The quadratic symmetric structure can further optimize the stress distribution through higher-order symmetry, which enables the patch to evenly distribute the load in multiple directions and reduce the stress concentration [29]. Under the control condition of quarter symmetry, the results for 10–40 % weight reduction show that the system still retains a relatively large amount of material despite the initial weight reduction (Fig. S4). A 50–80 % weight reduction shows that the structure becomes more simplified, and the symmetry is more pronounced. At 70–80 % reduction, the optimized morphology shows an almost circular or symmetrical quadrangular structure, with most of the areas of retained material concentrated at the edges and in areas of geometric symmetry, forming a distinct frame morphology. Again, as with the previous group, at 90 % weight reduction, further weight reduction is beyond the feasibility of the structure as the system collapses and is unable to generate meaningful results.

The optimization results of the three conditions support the quadratic symmetric and semi-symmetric designs as they have more advantages in terms of structural stability and mechanical properties than the optimization results of the thickness control. The best compromise between mechanical requirements, printability and highest weight reduction was found with an 80 % weight-reduced structure (octagon) under quadratic symmetry conditions.

As scaffolds are essential components in tissue engineering, an increasing number of studies have focused on the rational design of scaffold architectures to enhance the repair and regeneration of target tissues [20,21]. Numerous examples can be found in the field of bone tissue engineering, where the porosity and pore diameter of scaffolds play a critical role in determining both mechanical properties and the rate of tissue regeneration [30]. Therefore, rational design of scaffold porosity, while ensuring adequate mechanical stiffness, is essential for achieving optimal tissue repair outcomes. Bone scaffolds, as typical three-dimensional structures, are generally designed using two main approaches: non-parametric and parametric methods [31]. Non-parametric design typically involves simple geometric units, such as diamond lattices, face-centered cubic (FCC) structures [32], body-centered cubic (BCC) and octahedral structures [33], as well as rhombic dodecahedrons for three-dimensional frameworks, and honeycomb patterns for two-dimensional planar configurations [34]. However, with the advancement of additive manufacturing (AM) technologies, these simple, non-parametric designs are increasingly insufficient to meet complex mechanical and biological requirements for clinical applications [35]. Consequently, parametric design—an umbrella term for algorithm-driven methods that generate geometries through adjustable parameters—has become the prevailing strategy [21,29]. Topology optimization, as a powerful parametric design method, was employed to systematically explore scaffold architectures that balance mechanical performance and weight reduction. Ideally, this approach ensures sufficient mechanical strength to meet clinical abdominal wall repair requirements, while limiting polymer mass, thereby reducing the amount of the associated acidic by-products released during degradation.

### 3.2 From design to print: optimization of the printing path

Building upon the simulation results, it is crucial to evaluate the manufacturability of the optimized design by assessing its compatibility with practical printing path algorithms. Achieving a continuous and non-redundant printing path is vital for minimizing material waste and enhancing production efficiency. To address this, different path planning algorithms were applied to the selected structure, and their performance was systematically analyzed.

As shown in Fig. 1B, simulation result (Octagon), which possesses symmetry and is suitable for the research objective of printing path optimization, consists of 12 nodes and 16 edges. Based on this structure, four different path planning algorithms were explored: TCP (Traveler's Problem Path) [36], LP (Longest Path Algorithm) [37], HC (Hamiltonian Path) [38], and CPP (Chinese Postman Path) [39]. The specific planning results of each path algorithm, as well as the repeated and missing edges of the paths, are listed in the accompanying table (Fig. 1B). Here, a “repeated edge” refers to an edge that is traversed more than once by the same algorithmic path, whereas a “missing edge” denotes an edge that is not visited at all.

The results of the four path planning algorithms reveal distinct advantages and limitations. TCP effectively visits all nodes but introduces three duplicate edges while omitting four edges, highlighting its inefficiency in edge coverage. LP and HC avoid duplicates but fail to cover four edges, limiting their suitability for complete path traversal. CPP ensures full edge coverage through four duplicate edges, leading to redundancy in the path. From a MEW standpoint, avoiding repeated deposition paths within the same layer helps prevent local material buildup, fiber thickening, and pore occlusion, thus maintaining uniform fiber distribution and mechanical consistency [40,41]. Furthermore, simulation result (Octagon) does not satisfy the conditions for a Eulerian circuit, making it impossible to print the structure in a single, non-repeating pass. This limitation increases the complexity of the printing process and contributes to structural inconsistencies and inefficiencies.

To further explore the optimization of the print path, a new combined structure was constructed by connecting four simulation results (Octagon), resulting in a structure with 33 nodes and 52 edges (Fig. S5A). Compared to a single basic unit, this combined structure increases complexity to explore more possibilities for identifying an optimal print path. In addition to applying the previously analyzed path planning algorithms (Fig. S5B), layered printing (Fig. S5C) was introduced as a strategy for the combination of two basic units, and the results of all methods are summarized in Fig. S5D. The results show that none of the six methods can achieve fully optimized path planning: TCP and CPP introduce 17 and 10 duplicate edges, respectively, although they cover all edges; LP and HC avoid duplicates but omit as many as 19 edges; and the layered printing methods M1 and M2 do not eliminate path redundancy completely, despite the reduction in the number of duplicate edges (6 and 4, respectively). The above results reflect the inherent limitations of the current structure, which does not satisfy the Eulerian loop condition, resulting in print paths that cannot be both omission-free and duplication-free at the same time. Moreover, the paths of these algorithms have many inflection points, which results in an overall instability of the printed scaffold and a low molding accuracy (Fig. S5E). This limitation of path planning significantly increases the complexity of printing and affects the printing efficiency and structural homogeneity.

To overcome the limitations of the original structure in path planning, an optimized design for the base unit was developed. As shown in Fig. 1C, diagonal connections were introduced to create a tessellated octagrid —an octagon-based repeating unit that is tessellated into a continuous grid and incorporates both orthogonal and diagonal struts to maximize node connectivity. The new structure was further assembled into a larger grid by repeating the optimized base units, enabling more efficient and scalable printing path planning. The TO design radically reshapes the connectivity of the structure by integrating diagonal connections into the base structure. Diagonal connections ensure that every node within the units is well connected, minimizing isolated edges and allowing for smoother transitions between paths. At the same time, by increasing internal connectivity, the structure reduces the need for duplicate edges when planning complete coverage paths. To further optimize the printing process, the structure was divided into four hierarchical layers for path planning:

① **Layer-to-layer Orientation**: Each layer consists of a set of parallel, non-overlapping paths. These layers are printed sequentially, ensuring complete coverage of the structure while maintaining a straightforward path design.
② **Simplified Path Complexity**: By reducing the complexity of each layer to simple straight lines, the hierarchical strategy eliminates the need for intricate path planning algorithms, which are prone to inefficiencies in complex structures.
③ **Non-Repetitive Paths**: The hierarchical approach ensures that all paths are unique and non-repeating across layers, effectively resolving the repetition challenges seen in traditional algorithms.

At this stage, we successfully developed an optimized structure and corresponding path that could be implemented by MEW. To ensure the reproducibility of the optimized topology, the final structure was re-evaluated through an additional round of topology optimization using the same simulation parameters and boundary conditions as the initial quarter-symmetry model. The results showed no further material removal, confirming the structural stability and convergence of the design (Fig. S1D). Building upon our previous studies [16], we design the basic unit with a nominal side length of 500 μm and pore spans of 200 μm (Space 1) and 150 μm (Space 2). SEM and optical microscopy revealed actual pore spans of 204.5 ± 9.9 μm and 150.9 ± 8.7 μm, and side length of 507.8 ± 8.3 μm, thereby demonstrating high architectural fidelity. The scaffold was fabricated using MEW with a fiber filament diameter of 10.21 ± 0.84 μm (Fig. 1D).

Although the present work targets abdominal-wall reconstruction, the combined workflow of topology optimization and MEW is inherently generic and can be readily adapted to other soft-tissue interfaces. Because topology optimization simply requires the re-definition of load cases and boundary conditions, geometries can be recalculated for tissues with different mechanical environments. MEW is particularly well suited for fabricating such thin, membrane-like scaffolds, and by integrating topology optimization, scaffold designs can be tailored to replicate a target tissue's mechanical profile—for instance, matching the nonlinear compliance of vessel walls or the anisotropic, J-shaped stress–strain response of heart valve leaflets [42]. Similarly, in peripheral nerve repair, combining topology optimization with MEW can yield nerve guidance conduits with specifically tailored mechanical properties and aligned topographical cues, thereby providing enhanced mechanical stability and promoting directional axonal regeneration [43]. This suggests that the MEW–topology optimization strategy can be broadly applied across diverse soft tissues, with structural optimization used to impart site-specific mechanical functionality. Moreover, the approach could be extended to other bioresorbable polymers beyond PCL, allowing customization of scaffold degradation profiles as needed.

### 3.3 Mechanical properties

While the optimization process and hierarchical printing strategy demonstrated the feasibility of achieving efficient and accurate path planning, it is crucial to ensure that the TO scaffold possesses the desired mechanical properties to meet practical application requirements. To this end, tensile testing was conducted to evaluate the scaffold's strength, stiffness, and deformability, providing critical insights into its structural integrity and suitability for biomedical applications.

**Fig. 2:**
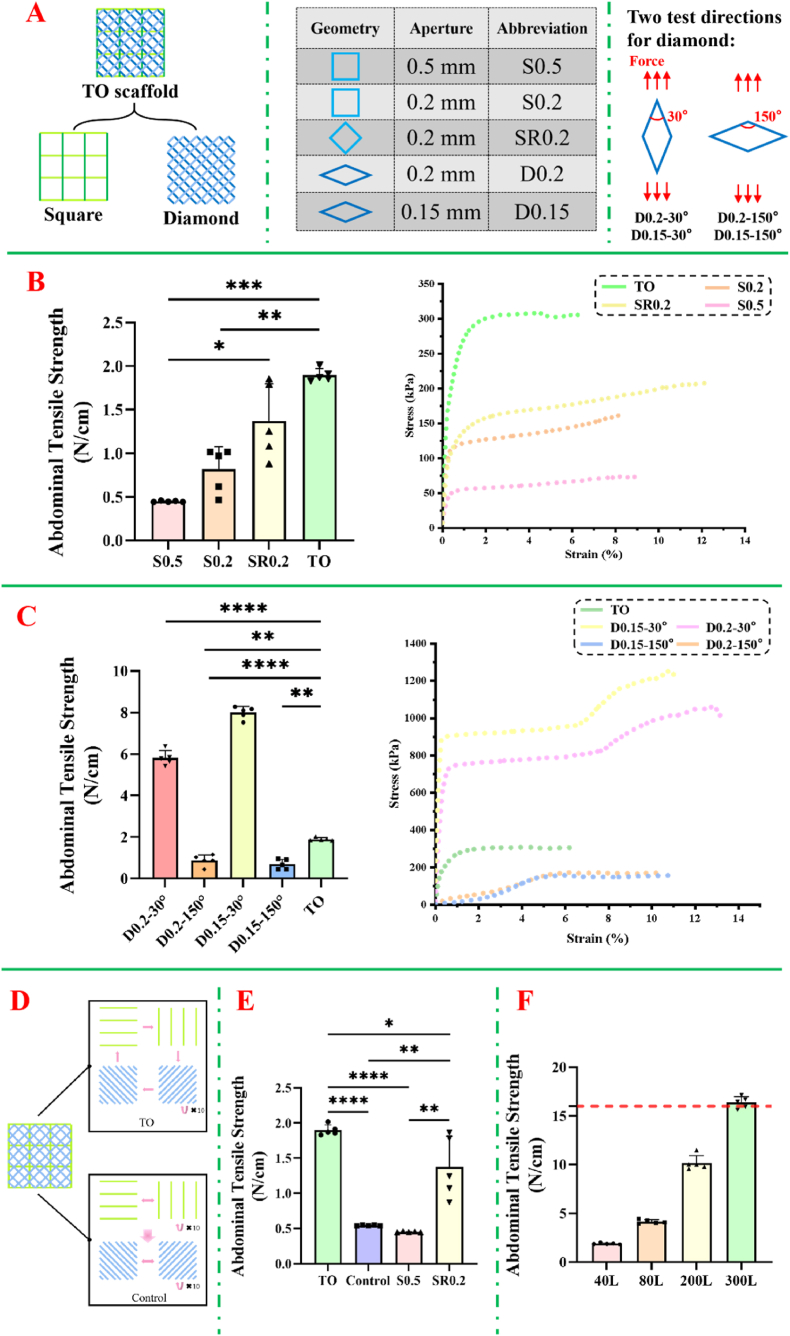
**Mechanical properties of TO scaffold designs**. (A) Scaffold configurations and loading directions. (B) Tensile strength and stress-strain curves of four different groups. (Abdominal wall tensile strength is expressed in N/cm, as patch thickness is negligible in mechanical assessments; n = 5) (C) Tensile strength under different loading directions (n = 5). (D) Layered printing strategies for TO and control groups. (E) Mechanical strength comparison between control and TO scaffold (n = 5). (F) Effect of layer number on mechanical performance (n = 5).

To systematically evaluate the mechanical properties of TO scaffolds, we compared it against several representative geometries and aperture sizes commonly considered for abdominal wall scaffolds (as shown in Fig. 2A). The first three groups (S0.5, S0.2, SR0.2) were chosen based on aperture dimensions similar TO scaffolds, reflecting typical mesh sizes and shapes (square, rhombus) that the TO print path includes. Additionally, the last two groups (D0.2, D0.15) were used to further investigate the multi-directional mechanical performance of TO scaffolds. Tensile tests were conducted in both vertical and horizontal directions.

As shown in Fig. 2B, the TO scaffold achieved a maximum stress value of 300 kPa, significantly surpassing the SR0.2 (200 kPa), S0.2 (150 kPa), and S0.5 (50 kPa) scaffolds. Additionally, the initial slope of the TO's curve was the steepest, indicating its superior stiffness compared to the other scaffolds. Quantitative analysis further revealed that the TO scaffold exhibited the highest abdominal wall tensile strength at 1.85 ± 0.02 N/cm, which was significantly greater than that of the S0.5, S0.2, and SR0.2 scaffolds (Table 2). In contrast, S0.5's simple rectangular pattern and large pore size contributed to its weakest mechanical performance. Although S0.2 and SR0.2 used smaller apertures that typically enhance strength, however, they still fell short of the TO scaffold, underscoring the advantages of topology simulation and geometric optimization in improving mechanical properties.

**Table 2:** Mechanical test results of different scaffolds. (n = 5).

Scaffold	Abdominal tensile strength (N/cm)
TO	1.85 ± 0.02
S0.5	0.45 ± 0.01
S0.2	0.82 ± 0.31
SR0.2	1.33 ± 0.51
D0.15–30°	7.99 ± 0.41
D0.2–30°	5.82 ± 0.50
D0.15–150°	0.69 ± 0.25
D0.2–150°	0.88 ± 0.38

To further validate the mechanical performance of the TO scaffold, 150° diamond-shaped units were tested. Smaller apertures of 200 μm and 150 μm were utilized, with tensile tests conducted in two loading directions to comprehensively evaluate the impact of geometry and aperture size on performance (Fig. 2C). The results are showed in Table 2, the D0.15–30° scaffold exhibited the highest tensile strength, followed by the D0.2–30° scaffold, demonstrating superior mechanical performance under 30° loading due to optimized fiber alignment and stress distribution. In contrast, the D0.15–150° and D0.2–150° scaffolds showed significantly lower tensile strength, indicating strong directional dependence. The TO scaffold displayed a moderate tensile strength (1.85 ± 0.02 N/cm) but maintained consistent performance across different orientations, highlighting its isotropic mechanical properties. While diamond-shaped scaffolds excel in specific loading directions, their directional sensitivity limits their adaptability in abdominal wall repair, where multi-directional stress resistance is crucial [10]. In comparison, the TO scaffold, with its optimized geometry and larger pore size, offers a more uniform mechanical response, making it better suited for practical applications.

Although the geometry of the TO scaffold was derived through topology optimization, and a balanced printing hierarchy was achieved in the optimized print path, its final morphology resembles a conventional mesh structure. This questions whether the originally optimized octagonal features are preserved and actively contribute to its mechanical properties. As shown in Fig. 2D, the primary objective of this experimental design is to verify the effectiveness of the topologically optimized structure in the final printed scaffold. Despite the simplification of the print path, resulting in a mesh-like appearance, it is hypothesized that the structural advantages of the TO scaffold still derive from the stress dispersion and multidirectional support capabilities inherent to the octagonal geometry. To address this, a comparative experiment was conducted between the TO scaffold and a control group. In the control group, a distinct printing strategy was employed, dividing each layer's printing into two parts, resulting in a scaffold that is partially rectangular and partially rhombic. Both groups were designed with identical layer counts to ensure comparable experimental conditions. The experimental results are presented in Fig. 2E. The TO scaffold demonstrated the highest tensile strength, significantly outperforming all other groups (p < 0.0001). The control group exhibited a tensile strength of 0.54 ± 0.07 N/cm, which was markedly lower compared to the TO group (p < 0.0001). The S0.5 scaffold displayed the lowest tensile strength among the four types, while the SR0.2 scaffold showed an intermediate value, higher than the control (p < 0.01), but still below that of the TO group. Therefore, the TO scaffold retains the octagonal geometry derived from topology optimization. Although the control group and TO scaffold use the same material and total number of layers, the control group lacks the well-structured print path present in the TO scaffold, resulting in insufficient geometric support between layers. This inhomogeneity significantly reduces its tensile properties. Conversely, the superior performance of the TO scaffold further confirms that the topology-optimized geometry is effectively retained in the final structure, contributing to enhanced mechanical properties.

Furthermore, an optimized layered scaffold structure prevented stress concentration and preserved mechanical integrity, compared to a control group with identical materials and layer counts. To systematically evaluate the relationship between layer number and mechanical performance, we designed a series of scaffolds with varying layer numbers (40L, 80L, 200L, and 300L) and measured their abdominal tensile strength. As shown in Fig. 2F, the abdominal tensile strength increased with layer number. Finally, achieving the 16 N/cm clinical benchmark for abdominal wall repair required at least 300 layers, but the thin fiber diameter prevented excessive overall thickness, meeting practical clinical needs.

Other abdominal patch design methods typically rely on simple geometric pores (e.g., the S0.5, S0.2, and SR0.2 groups in our study) [10], but as shown in Fig. 2C, these non-parametric structures are mechanically inferior to the topology-optimized scaffold, highlighting the benefits of algorithm-based optimization. In terms of fabrication, FFF-printed PCL patches meet strength requirements; FFF-printed PCL patches readily satisfy the tensile-strength requirement; however, their filaments are typically >200 μm in diameter and generate pores of ∼500–1000 μm [44]. Such coarse strands, combined with the polymer's inherent hydrophobicity, impede cell adhesion and tissue integration, potentially exacerbating postoperative discomfort [45,46]. Conversely, electrospun PCL membranes display excellent porosity and biocompatibility for guiding cell proliferation, yet the nanofibers they produce (≈100–500 nm in diameter) [47] lack the load-bearing capacity and positional precision needed to optimize scaffold-level mechanical performance [48,49]. In contrast, the MEW technique, which offers micron-scale fiber diameters and high-resolution design, enables the production of PCL patches that not only meet the clinical tensile strength standard (>16 N/cm) but also maintain superior flexibility. Although MEW processing parameters are well established [13], this study did not systematically explore design parameters. MEW can produce fibers from 2 to 50 μm in diameter [50], and increasing the diameter by only 5 μm has been shown to enhance inter-layer bonding by ∼70 % [51], implying that our current 10 μm filaments may not be mechanically optimal. Future work will therefore co-optimize fiber diameter and layer count to meet strength requirements while shortening fabrication time and enhancing the scaffold's clinical applicability.

### 3.4 Biocompatibility analysis of TO Scaffolds

#### 3.4.1 Evaluation of adhesion and cell proliferation on TO Scaffolds

To streamline the preparation process and specifically investigate how the TO scaffold influences cell distribution and growth orientation, all scaffolds used in the cell experiments were prepared with a four-layered structure, representing the smallest repeating unit of the TO scaffold. Additionally, to evaluate the effect of surface treatment on biocompatibility, two comparative groups were included: pure PCL scaffolds (TO-P) and collagen-coated PCL scaffolds (TO-C).

**Fig. 3:**
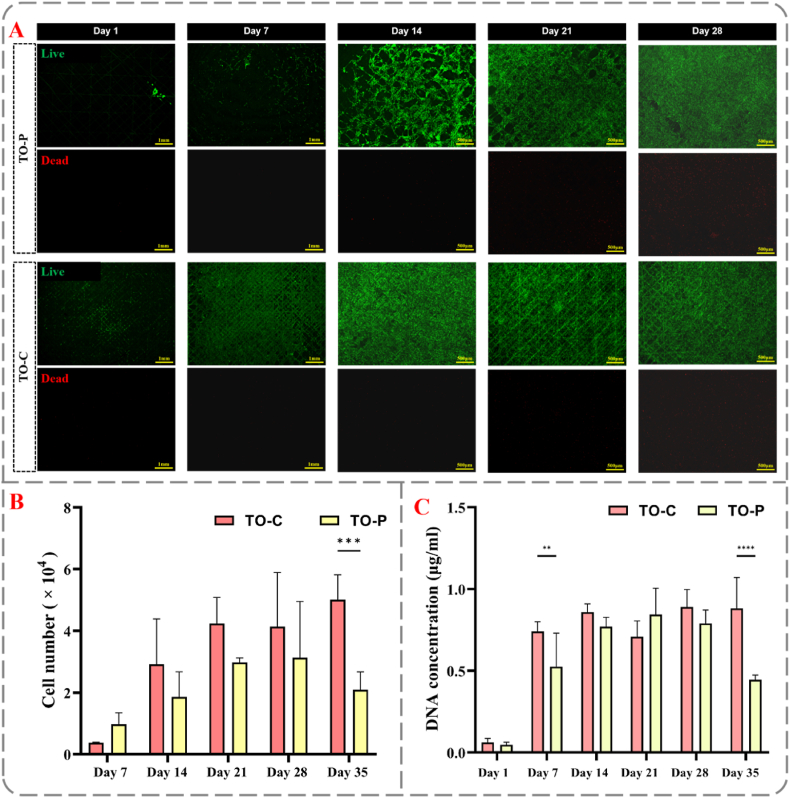
**Evaluation of NHDF proliferation on TO scaffolds**. (A) Live/dead staining of fibroblasts on TO-P and TO-C scaffolds. Fluorescent microscopy images show live (green) and dead (red) cell staining on TO-P (pure PCL) and TO-C (collagen-coated) scaffolds at different time points (day 1, 7, 14, 21, 28); scale bars represent 500 μm (day 1 & 7) and 1 mm (day 14–28), respectively. (B) Cell number quantification. Cell count over time on TO-C and TO-P scaffolds (∗∗∗p < 0.001). (C) Analysis of DNA content. DNA concentration on TO-C and TO-P scaffolds over time (∗∗p < 0.01, ∗∗∗∗p < 0.0001).

Fig. 3A presents the live/dead staining results of NHDF cultured on TO-P and TO-C scaffolds at different time points (days 1, 7, 14, 21, and 28). Green fluorescence represents live cells, while red fluorescence indicates dead cells. At day 1, a small number of cells were observed to attach to the TO-P scaffolds, whereas the TO-C scaffold exhibited more extensive green fluorescence, indicating greater and more uniform cell attachment. By day 7, cell proliferation was evident in both groups; however, the TO-P group displayed a sparser distribution, while the TO-C group showed improved proliferation and more extensive surface coverage. In addition, Fig. S6 provides almost full-surface live/dead staining of scaffold at day 7. And Fig. S6 B&C demonstrates that the TO-C supports a uniformly distributed cell layer, whereas TO-P remains sparsely populated. At day 14, cells on TO-P scaffolds began to cover most of the scaffold surface, whereas TO-C scaffolds were nearly fully covered with live cells. By day 21 and day 28, both groups had formed a continuous cell layer on the scaffold surface. Throughout the entire incubation period, no significant increase in dead cells was observed in either the TO-C or TO-P groups.

Fig. 3B presents the number of viable NHDF detected on TO-P and TO-C scaffolds using the LDH activity assay at various time points (days 7, 14, 21, 28, and 35). The TO-C group (red) consistently exhibited higher numbers of viable cells across all time points, which was significantly higher compared to the TO-P group (p < 0.001). The TO-P group (yellow) consistently showed lower numbers of viable cells, with the most pronounced differences observed on days 7 and 35. Fig. 3C depicts the changes in DNA content within TO-P and TO-C scaffolds over time (days 1, 7, 14, 21, 28, and 35). Both groups demonstrated a similar trend in DNA content compared to the outcome of the LDH measurement. DNA content was significantly higher for the collagen-coated scaffolds after 7 days of cultivation.

These observations align with our prior studies [14], wherein NHDF and stem cells were cultured on collagen-coated PCL fiber scaffolds. In these earlier studies, the cells demonstrated robust proliferation and migration capabilities, achieving complete coverage of the scaffold surface within one week. Furthermore, beyond the MEW process, PCL scaffolds fabricated through weaving [52] and 3D printing [53] techniques also show enhanced cell growth when coated with collagen. This observation underscores the pivotal role of collagen in facilitating cell interactions within synthetic polymer matrices. Such enhancements are critical for promoting the cellular functions essential for successful tissue engineering applications.

#### 3.4.2 Characterization of fibroblast morphology and orientation on TO Scaffolds

To gain deeper insights into cell morphology and cytoskeletal organization, the nucleus (DAPI, blue), F-actin cytoskeleton (Phalloidin, green), and intermediate filaments (vimentin, magenta) were stained for fluorescence microscopy, enabling a comprehensive assessment of cell-scaffold interactions. Especially, to quantitatively evaluate cell alignment and distribution patterns on the scaffold surface.

**Fig. 4:**
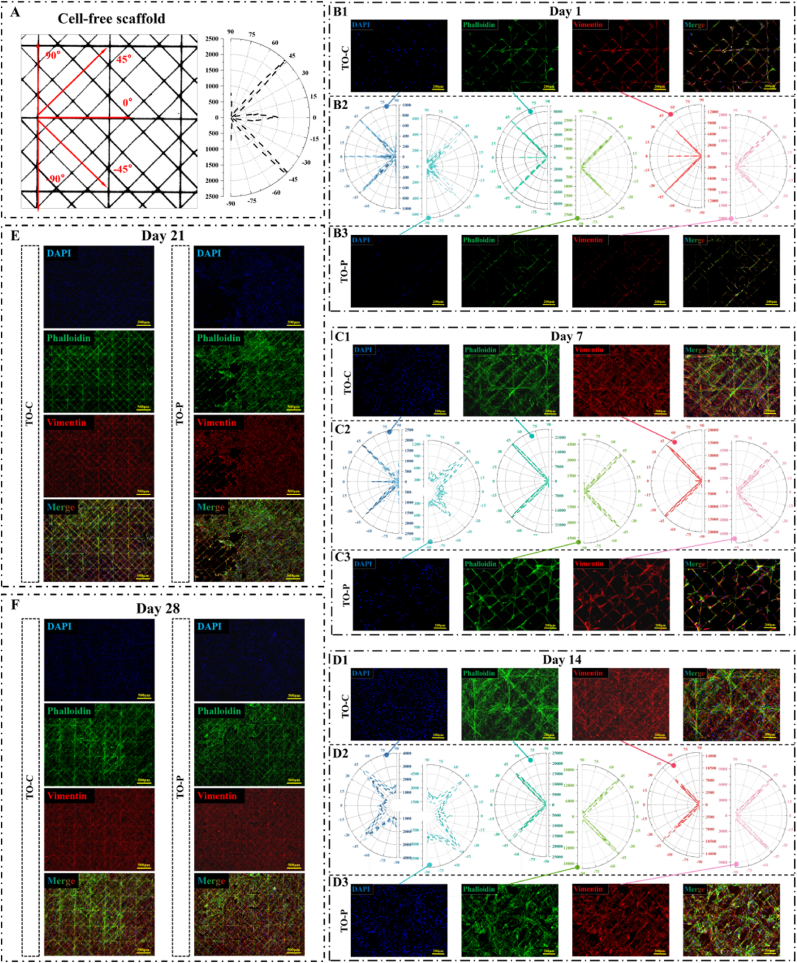
**Evaluation of cell orientation and cytoskeletal organization on TO Scaffolds**. (A) Cell-free scaffold analysis. Diagram of the cell-free TO scaffold showing defined orientation angles (0°, 45°, −45°, 90°) used as references for cellular alignment analysis. Polar plot depicts fiber orientation counts. (B1-B3) Day 1, (C1-C3) day 7, (D1-D3) day 14, (E) day 21, (F) day 28. Live cell staining of nuclei (DAPI, blue), F-actin cytoskeleton (phalloidin, green), and intermediate filaments (vimentin, red) on TO-C (collagen-coated) and TO-P (pure PCL) scaffolds over time. The merged images demonstrate cellular distribution and alignment within the scaffold (n = 3). (B2, C2, D2). Polar plots of cell orientation: Quantitative orientation analysis of cells stained with DAPI, phalloidin, and vimentin on TO-C scaffolds at different time points (day 1, 7, 14). Scale bars: 200 μm for B1–B3, C1–C3, and D1–D3; 500 μm for E and F.

Fig. 4A shows the analysis of the cell-free TO scaffold, establishing a baseline for studying cellular alignment. In the left panel, key fiber directions are indicated: 0° (horizontal), 90° (vertical), and ±45° (diagonal). The right panel is a polar plot quantifying fiber density along these orientations.

At day 1 (Fig. 4B1&B3), DAPI staining on the TO-C scaffolds indicates a greater number of nuclei, while phalloidin and vimentin staining reveal the early establishment of actin filaments and intermediate filaments aligned with the scaffold fibers. In contrast, the TO-P scaffolds show fewer attached cells, limited cytoskeletal formation, and weaker orientation (Fig. 4B2). By day 7 (Fig. 4C1&C3), the TO-C scaffolds are densely populated with cells exhibiting well-developed actin networks and intermediate filaments, whereas the TO-P scaffolds, although showing improved cellular presence, remain less uniformly covered. The corresponding polar plots (Fig. 4C2) confirm that, in the TO-C group, cells continue to align along four main directions (0°, 45°, −45°, and 90°), whereas the TO-P scaffolds reveal more limited cell orientation.

By day 14 (Fig. 4D1&D3), the TO-C scaffolds display even greater cell density with extensive cytoskeletal alignment, particularly in diagonal orientations. Although the TO-P scaffolds also show increased cell numbers, their distribution and cytoskeletal maturity remain less uniform than that seen in TO-C. The polar plot analyses (Fig. 4D2) indicate a marked rise in cell alignment strength in both groups, but TO-C continues to show a more balanced coverage. At day 21 and 28 (Fig. 4E and F), both groups exhibit nearly complete cell coverage, forming dense, continuous layers with mature cytoskeletal features—phalloidin staining confirms uniform F-actin extension, while vimentin indicates well-established intermediate filaments throughout the scaffold. By these later stages, the differences between the TO-C and TO-P groups diminish, with both appearing highly adapted to their respective scaffold environments.

The results show that the TO scaffold's intrinsic geometry significantly influences cell attachment, orientation, and proliferation. Its aligned fiber arrangement provides directional cues that guide early cell growth, as confirmed by phalloidin and vimentin staining, with the TO-C group exhibiting particularly organized cell alignment. This work is consistent with our previous findings. Von Witzleben et al. employed MEW to fabricate PCL scaffolds coated with collagen for replacement of the tympanic membrane, observing similar trends in fibroblast proliferation and functional enhancement [15]. MEW-based scaffolds are widely recognized for their ability to guide cellular growth, and have been used to align corneal cells [54], periodontal fibroblasts [55], and hMSC [56]. However, most MEW scaffolds reported in the literature employ diamond-like networks at varied angles or variations in fiber spacing and diameter to induce predominantly unidirectional cell alignment [57,58]. In contrast, TO scaffold was optimized based on a symmetric square topology, leading to a distinct cell orientation pattern along both 0°/180° and ±45°. This multi-directional alignment promotes more uniform tissue formation upon cell coverage, aligning well with the multi-axial stress distribution characteristic of the abdominal wall, thereby enhancing its suitability for clinical application.

#### 3.4.3 Cellular response to high-layer TO Scaffolds for abdominal wall applications

Up until now, we focused on the cellular interaction with the four-layer TO scaffold structure. However, given that the final TO scaffolds intended for abdominal wall patch applications need to meet more stringent mechanical requirements, subsequent experiments utilized a 300-layer scaffold (Fig. 2F). This approach aims to simulate conditions closer to clinical application and allows for a more comprehensive evaluation of the biological performance of scaffolds at greater thickness. SEM micrographs in Fig. S7A and B reveal minor filament misalignment in the 300-layer TO scaffold due to charge build-up during MEW; nevertheless, quantitative measurements (Fig. S7C) show that fiber diameter and pore size remain within the intended design range, confirming that architectural fidelity is maintained even after scaling to 300 layers.

**Fig. 5:**
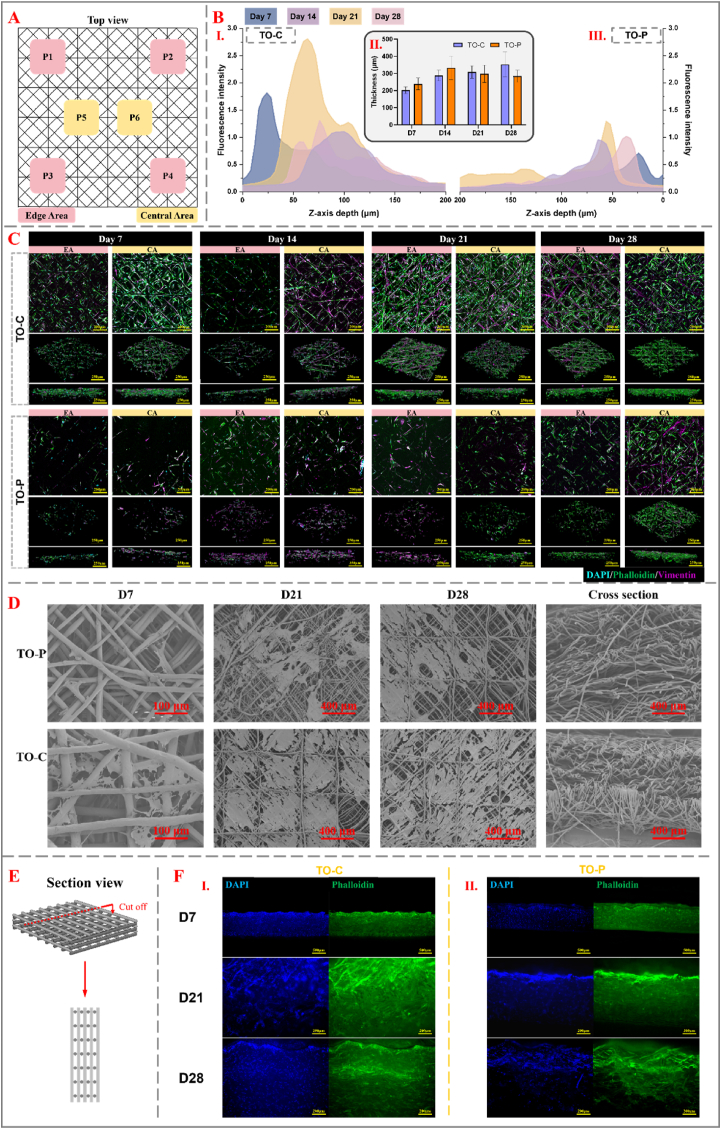
**Cellular Distribution and Penetration Analysis of TO Scaffolds Over Time**. (A) Top view of cell seeding locations. Diagram showing the edge area (EA, P1-P4) and central area (CA, P5-P6) used for analysis of cell distribution and migration on TO scaffolds. (B) Fluorescence intensity analysis along the Z-axis. I & III: Fluorescence intensity of TO-C and TO-P scaffolds. Z-axis depth analysis of fluorescence intensity representing cellular penetration across different depths (0–200 μm) at various time points (day 7, 14, 21, 28); II: Depth of cell penetration. Bar graph showing the average penetration depth of cells (TO-C and TO-P) at different time points. (C) Confocal microscopy analysis of TO-C and TO-P scaffolds. Representative images showing DAPI (cyan), phalloidin (green), and vimentin (magenta) staining in the edge (EA) and central (CA) regions of the TO-C and TO-P scaffolds at day 7, 14, 21, and 28. The images indicate cell attachment, orientation, and three-dimensional expansion; scale bars represent 250 μm. (D) SEM of 300-layer scaffolds. Rows: TO-P vs TO-C; columns: day 7, 21, 28, and cross-section. (E) Illustration of a cross-sectional view of a TO scaffold used for permeability analysis. (F) Cross-sectional fluorescence imaging of cell penetration. I: TO-C scaffold; II: TO-P scaffold. DAPI (nuclei) and phalloidin (F-actin) staining on day 7, 21, and 28, showing substantial cell penetration and scaffold infiltration (scale bars: D7 500 μm; D21&28 200 μm).

To evaluate the spatial distribution of cells across the TO scaffold, both within the plane and through the scaffold's thickness, the experiment involved strategic seeding of NHDF cells in the central region of the scaffold. As illustrated in Fig. 5A, the scaffold was divided into two key regions for analysis: the central area (CA) and the edge area (EA). Specifically, two central points (P5 and P6) were selected to represent the central region, while four peripheral points (P1, P2, P3, and P4) were used to characterize the edge area.

Fig. 5C shows the cell growth on the edge area (EA) and central area (CA) of the scaffolds of two groups at day 7, 14, 21, and 28. On day 7, the TO-C group exhibited greater cell attachment in both the EA and CA regions, with higher cell density and a more uniform distribution compared to the TO-P group. Phalloidin and vimentin staining revealed that cytoskeletal and intermediate filaments extended along the scaffold fibers, though overall cell coverage remained limited. By day 14, a significant increase in cell density was observed in both the EA and CA regions of the TO-C group, particularly in the CA region, where coverage was notably higher. Phalloidin and vimentin staining demonstrated that the cytoskeleton and intermediate filaments expanded considerably in all directions, including a deeper penetration along the Z-axis, indicating robust three-dimensional cell expansion. On days 21 and 28, the TO-C scaffold was nearly fully covered by cells, forming a dense, continuous layer across its surface. Z-axis images also demonstrated substantial cell penetration, indicating successful cellular integration within the scaffold. In contrast, the TO-P group consistently demonstrated weaker cell attachment and expansion at all observed time points. On day 7, cell density in both the CA and EA regions of TO-P was lower and more sparsely distributed, with phalloidin and vimentin staining indicating limited cytoskeleton and intermediate filament formation, lacking significant orientation. By day 14, although cell numbers increased, the coverage of CA and EA regions remained notably lower than that of the TO-C group, with insufficient cytoskeletal extension and connectivity. On days 21 and 28, cell coverage in TO-P increased further but still lagged the TO-C group in terms of overall distribution and uniformity. Notably, Z-axis analysis showed less cell penetration, indicating suboptimal three-dimensional cellular integration and extension. Moreover, the SEM micrographs in Fig. 5D demonstrate that TO-C has higher initial cell attachment than TO-P and progressively achieve near-complete coverage of the scaffold with time. The pronounced extracellular-matrix build-up on TO-C further corroborates the superior surface coverage observed in Fig. 5C.

Fig. 5B shows the main distribution areas and the penetration depth of cells into the scaffold. In the TO-C group (Fig. 5BⅠ), the fluorescence intensity progressively increased over time (days 7, 14, 21, 28), indicating enhanced cell proliferation and penetration. At day 7, cells were predominantly localized near the scaffold surface (<50 μm), reflecting limited initial penetration. By day 14, the fluorescence signal significantly increased and extended deeper, with notable intensity observed up to approximately 100 μm depth, suggesting active penetration into the scaffold interior. At days 21 and 28, cells displayed further deep-layer distribution, extending between 50 and 150 μm, indicating effective utilization of the scaffold's three-dimensional structure and enhanced cellular expansion along the Z-axis. In Fig. 5BIII, representing the TO-P group, the fluorescence intensity over the Z-axis depth shows a much lower overall intensity compared to the TO-C group. Most of the cells remained within the superficial region (<50 μm) across all time points, with minimal expansion into deeper layers, even by day 28. In Fig. 5BII, the depth of cell penetration into the scaffold (represented as thickness) was analyzed for both the TO-C and TO-P groups at different time points (days 7, 14, 21 & 28). The results show that the penetration depth of cells was comparable between the two groups throughout the entire observation period, with no significant differences in the measured thickness values. However, despite similar penetration depths, the cell density differed substantially. This observation, when compared with the fluorescence intensity profiles, suggests that while some cells from the TO-P group did migrate to deeper regions, the overall cellular density was much lower compared to the TO-C group. Essentially, the TO-P scaffold showed lower cellular coverage in deeper layers, indicating that while individual cells might reach these depths, the proliferation and uniform distribution were not as robust as in the TO-C scaffold.

To further verify the cellular infiltration into the scaffold, the TO scaffold was sliced perpendicular to the fiber layer with a scalpel as illustrated in Fig. 5E. In the TO-C group (Fig. 5FⅠ), the DAPI signal demonstrated clear and extensive cell penetration throughout the scaffold depth from day 7 to day 28, suggesting effective cell infiltration and growth in the interior layers. The phalloidin staining showed a continuous cytoskeletal formation across the scaffold, with increasing density over time, reflecting robust cellular expansion and attachment. In contrast, the TO-P group (Fig. 5FⅡ) exhibited a comparatively lower cell penetration depth and cytoskeletal extensions. At all-time points, DAPI staining revealed less uniform cellular distribution, and phalloidin staining indicated a more restricted extension of the cytoskeleton, particularly evident at day 28. These findings suggest that, although some cells did infiltrate the deeper layers of the TO-P scaffold, the overall cellular growth and integration were not as effective as observed in the TO-C scaffold. Similarly, SEM cross-sections in Fig. S7D**1–E2** show that by days 21 and 28 the TO-C scaffold is enveloped by a continuous, sheet-like cell/ECM layer interwoven with the fibers, evidencing substantial Z-axis migration. In contrast, the TO-P scaffold exhibits only sparse surface coverage (Fig. S7F1, F2).

In the current study, our 300-layer scaffold—engineered to meet mechanical requirements for abdominal wall repair—offered a substantially thicker three-dimensional environment that effectively supported cell infiltration under static culture conditions. This finding aligns with prior reports on large-volume MEW scaffolds, where multi-layer architectures and precise fiber arrangements have been shown to facilitate deeper cellular penetration [41,59]. For instance, Wunner et al. [41]and Hrynevich et al. [59] demonstrated that by controlling parameters such as fiber spacing and layer stacking, significantly thicker MEW constructs maintain sufficient porosity for sustained nutrient diffusion and cell migration. Building on these insights, our future work will priorities further increasing fiber surface area and overall porosity—while still meeting all mechanical benchmarks—to lower polymer content, shorten fabrication time, and enhance Z-axis cell migration thereby improving clinical translational potential.

Moreover, our study employed a static culture method and demonstrated that collagen coating (TO-C) significantly enhanced early-stage cell adhesion and proliferation. However, existing literature [60] suggests that under dynamic culture conditions, such as in perfusion bioreactor systems, scaffolds may exhibit improved cell penetration along the Z-axis. This enhancement is likely due to the dynamic fluid environment, which facilitates the efficient transport of cells into the deeper regions of the scaffold. Additionally, as collagen coatings may slightly reduce pore openness, potentially restricting initial cellular infiltration, uncoated scaffolds (TO-P) could exhibit superior early-stage penetration under dynamic conditions. Nevertheless, in the context of *in vivo* implantation, cell migration plays a more pivotal role than initial seeding efficiency [61]. Collagen coatings contribute to sustained cellular infiltration and integration by providing additional adhesion sites and bioactive cues, which actively support deeper cell migration and tissue formation over prolonged culture periods. Thus, while dynamic culture may initially enhance the infiltration of uncoated scaffolds, collagen-coated scaffolds retain a critical advantage in thicker constructs, as they promote long-term cellular migration, stabilization, and integration within the scaffold. Another limitation of this study is the absence of direct quantification of coating homogeneity within the 300-layer scaffold. The almost full-field live/dead images in Fig. S6 B&C and our previous study [62] confirm uniform collagen coverage only in thin MEW scaffolds. For the 300-layer scaffold, uniformity is inferred solely from the surface-level confocal images in Fig. 5C, leaving intraluminal penetration uncharacterized. Because the coating was applied by immersion, the Washburn capillary-flow model predicts a marked decline in penetration depth with increasing diffusion path length and pore resistance [63]. Consequently, the multilayer MEW patch with micron pore size may harbor a central zone with insufficient coating, potentially resulting in heterogeneous cell distribution and local mechanical responses.

### 3.5 Influence of cellular infiltration on TO Scaffold biomechanics and ex vivo feasibility validation

#### 3.5.1 Biomechanical testing of TO Scaffolds post-cellular integration

Building on the observed advantages of collagen coating in enhancing cell attachment and proliferation from previous cellular experiments, this part aimed to further investigate the impact of cell attachment on the mechanical properties of TO scaffolds. Specifically, biomechanical tests were conducted on TO scaffolds that had been separately cultured with cells, including the TO-P (pure PCL) and TO-C (collagen-coated) groups. After culturing cells on the scaffolds for a predetermined period, tensile tests were performed to evaluate how cell attachment and proliferation influenced the overall mechanical characteristics of the scaffolds.

**Fig. 6:**
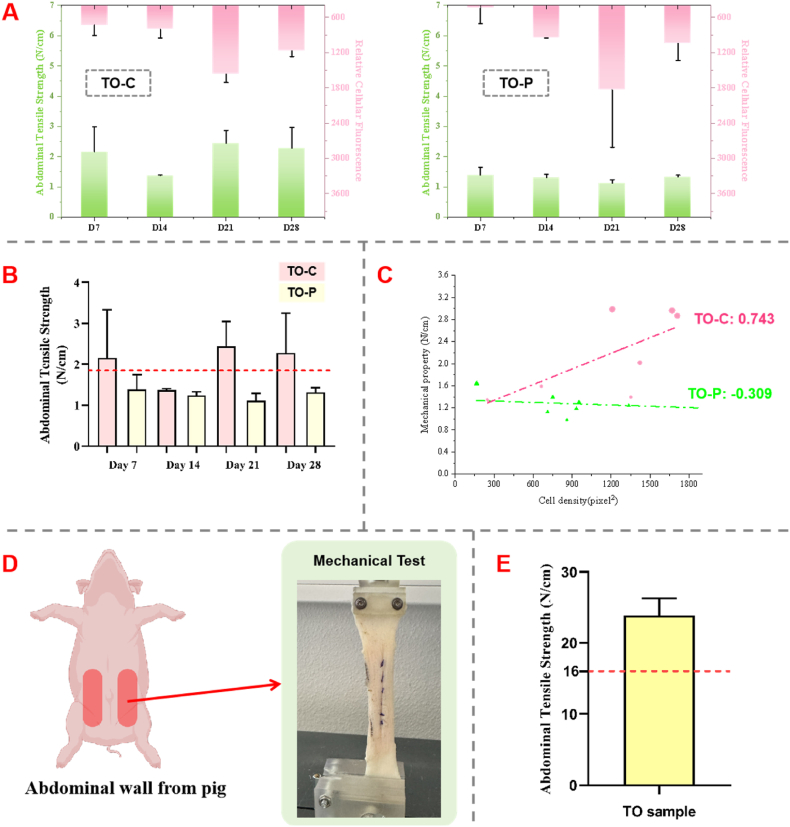
**Evaluation of Mechanical Properties of TO Scaffolds with Cellular Integration and *Ex Vivo* Testing**. (A) Biomechanical properties and cell proliferation of TO-C and TO-P scaffolds. Bar charts showing the tensile strength (green) and corresponding relative cellular fluorescence intensity (pink) of TO-C (left) and TO-P (right) scaffolds after culturing for different durations (day 7, 14, 21, 28) (n = 3). (B) Comparative tensile strength between TO-C and TO-P. Mechanical performance of TO-C and TO-P scaffolds at various time points. The dashed red line represents the tensile strength of acellular TO-C scaffolds. (C) Correlation analysis of cell density and mechanical strength. Scatter plot showing the correlation between cell density and mechanical properties for TO-C and TO-P scaffolds. (D) *Ex vivo* mechanical test setup. Schematic showing the area of the excised abdominal wall specimens from a pig (left) and the mechanical testing setup (right, the red dotted box is the location of the scaffold), whereby TO-C samples were sutured to the porcine tissue samples to evaluate the tensile properties. (E) Tensile strength of TO-C scaffolds in an *ex vivo* model. Bar graph representing the tensile strength achieved by TO-C scaffolds in the *ex vivo* porcine abdominal wall model. The dashed red line represents the benchmark of the abdominal wall tensile strength gold standard (16 N/cm) (n = 4).

Fig. 6A illustrates the trends in tensile strength (green) and cytofluorescence intensity (pink) for TO-C and TO-P scaffolds over different cultivation time points (day 7, 14, 21, 28). The cell fluorescence intensity was quantified through live/dead staining, with measurements taken at four distinct positions on each scaffold, and at three different heights for each position (Fig. S6A).

In the TO-C group (left panel), cytofluorescence intensity increased substantially with prolonged incubation, particularly between day 14 and day 28, indicating significant cellular proliferation – like the experimental results described above. Notably, the tensile strength initially dropped at day 14 before recovering by day 28, suggesting a dynamic interaction between cell growth and scaffold mechanics. Despite this fluctuation, cytofluorescence intensity and mechanics exhibited a generally similar trend over time. In contrast, the TO-P group (right panel) showed only minor increases in cytofluorescence, indicating slower cell proliferation. Fig. 6B illustrates the tensile strength of TO-C and TO-P scaffolds with attached cells at different time points (day 7, 14, 21, 28). The tensile strength of the TO-C group (pink) was consistently higher than that of the TO-P group (yellow) at each time point except day 14. Notably, the red dashed line represents the tensile strength of the scaffold without cells (∼1.85 N/cm). It can be observed that the tensile strength of the TO-C group surpassed that of the acellular scaffold at all points except day 14, suggesting a possible positive influence of cell attachment on scaffold mechanics. In contrast, the TO-P group maintained tensile strength levels similar to or below that of the acellular scaffold. Fig. 6C presents a scatter plot evaluating the correlation between cell density (as measured by pixel intensity) and tensile strength for both the TO-C and TO-P groups. The Pearson correlation coefficient was calculated to determine the relationship between these two variables. The TO-C group (red dots) demonstrated a positive correlation (r = 0.743) between cell density and tensile strength, indicating that increased cell proliferation may enhance the mechanical properties of the scaffold. On the other hand, the TO-P group (green triangles) displayed a weak negative correlation (r = −0.309), suggesting that cell proliferation had little to no effect on mechanical strength for this group, or may even have had a slightly adverse effect.

Although the TO-C scaffolds displayed a moderate positive correlation between cell density and abdominal tensile strength (Pearson r = 0.743), this increase cannot yet be attributed solely to the collagen coating. The effects of cell attachment and proliferation on the mechanical properties of TO scaffolds remains multifactorial, encompassing factors such as scaffold degradation [64,65] and collagen coating [66,67]. Accordingly, our future work will further examine how degradation characteristics and coating modifications affect mechanical performance, and will incorporate dynamic culture conditions to systematically evaluate the scaffold's mechanical properties under more complex environments. Nonetheless, the observed mechanical enhancement in collagen-coated scaffolds after 28 days of culture presents a promising outcome for long-term scaffold stability and functionality.

#### 3.5.2 *Ex vivo* validation of TO Scaffolds in porcine abdominal wall repair

For the final biomechanical assessment, we used an *ex vivo* porcine abdominal wall model to verify the functional performance of the TO-C scaffolds (300 layers) in a setting that simulates clinical application conditions. As shown in Fig. 6D, the scaffold (10 cm × 10 cm) was sutured to an artificially created 1 cm × 1 cm defect in the porcine abdominal wall, and tensile testing was performed to evaluate the scaffold's mechanical strength after incorporation with biological tissue. The other steps involved in creating, repairing, and testing that defect can be seen in Fig. S6B.

The results, presented in Fig. 6E, show that the abdominal tensile strength of the TO scaffold reached approximately 22 N/cm, which exceeded the clinically recommended abdominal wall repair standard of 16 N/cm (indicated by the red dashed line in the Figure). This suggests that the TO scaffold can not only interface effectively with biological tissue but also achieve a tensile strength surpassing the required minimal threshold for effective abdominal wall repair.

Compared to PCL patches fabricated using other methods (e.g., FFF & Extrusion printing) [45]and commercial polypropylene (PP) patches [68,69], MEW scaffolds feature finer fibers, reduced stiffness, and greater overall softness (Fig. S6B). Their superior flexibility and compliance after suturing may help reduce postoperative discomfort. Although PP meshes remain the clinical standard for hernia repair, their millimeter-scale monofilaments and high bending stiffness can impede tissue integration and provoke chronic inflammation [70]. Our MEW-printed PCL scaffold, by contrast, attains an abdominal tensile strength of 22 N cm^−1^—comfortably above the 16 N/cm guideline for abdominal-wall patches [4]—even though its ultimate strength is lower than that of heavy-weight PP [71]. Crucially, MEW produces very fine (∼10 μm) PCL fibers, orders of magnitude smaller than PP mesh filaments. The high surface‐to‐volume ratio and open pore network promote cell attachment, spreading and proliferation [72]. MEW scaffolds can also be surface‐modified (TO-C group) to further boost adhesion and integration. Unlike permanent PP meshes, which can provoke chronic granulomatous inflammation, the biodegradable PCL scaffold gradually transfers load to healing tissue [71]. These features fine architecture, tunable coatings and resorbability confer a distinct biological advantage to MEW PCL implants.

However, our current mechanical evaluations rely solely on static testing, which cannot fully replicate the complex dynamic loading conditions encountered *in vivo*, such as cyclic stresses arising from breathing, coughing, or muscle contractions. Although the *ex vivo* porcine abdominal wall model partially simulates the scaffold's interaction with native tissue under realistic static loading conditions, the actual clinical effectiveness, biocompatibility, and mechanical reliability under long-term dynamic loads remain to be validated. Therefore, animal models involving dynamic loading scenarios are essential to further optimize scaffold design and assess its functionality under realistic physiological conditions. Future work will incorporate *in vivo* models to further investigate scaffold degradation, immune response, and functional integration within abdominal tissues.

In addition, although MEW fabrication is somewhat more time-consuming compared to conventional patch production, the capital cost of the technology has fallen sharply: several commercial start-ups now market MEW modules that convert low-cost FFF printers into melt-electrowriting systems and recent advancements have significantly reduced MEW equipment expenses by upgrading standard 3D printers [73,74]. Consequently, hardware expenditure is no longer the dominant factor; the major cost drivers are GMP-compliant clean-room operation and skilled manpower for process validation and quality control. Material expenses remain comparable because most clinical patches—MEW or otherwise—use medical-grade PCL. Pre-fabrication with long-term sterile storage can offset MEW's lower throughput, while standardized scaffolds may be mass-produced and simply trimmed to patient-specific geometries. Taken together, current market developments indicate that MEW is economically viable, provided that labor and regulatory workflows are optimized.

Despite demonstrating excellent *in vitro* tissue conformability, MEW scaffolds may pose a risk of adhesion to visceral organs in clinical scenarios, potentially leading to complications such as organ tethering or obstruction—particularly within dynamic environments like the abdominal cavity. Moreover, abdominal wall patches require adequate antibacterial properties to mitigate infection risks during surgical procedures [10,11]. Future scaffold designs will therefore combine MEW with additional fabrication techniques, such as electrospinning of anti-adhesive fibrous membranes [75] and extrusion printing of antibacterial hydrogel layers [76], to comprehensively enhance scaffold performance. Furthermore, emerging dual-network hydrogel platforms enabling sustained delivery of bioactive proteins (e.g., IGF-1, BMP-7) [77,78] suggest that incorporating a growth factor-laden hydrogel or nanoparticle-laden interlayer into the topology-optimized MEW lattice could simultaneously reduce adhesion, provide antimicrobial or immunomodulatory cues, and augment regenerative capacity.

## 4 Conclusion

In this study, we systematically explored and optimized the design, as well as the mechanical and biological properties of a MEW scaffold for application to the abdominal wall. Initial topology optimization yielded a fine structure that enabled superior mechanical properties, and through further structural and pathway optimization, the TO scaffold was successfully prepared from PCL using MEW technology. Subsequent studies have shown that while both geometry and printing strategy affect the performance of the scaffolds, the collagen coating is critical in promoting early cell attachment and proliferation, especially in thicker (300-layer) scaffolds. Biomechanical testing showed that TO-C scaffolds displayed a moderate positive correlation (Pearson r ≈ 0.74) between cell density and tensile strength, and validation using an *ex vivo* porcine tissue model confirmed that TO scaffolds could meet the tensile strength requirements for abdominal wall repair. These findings suggest that TO scaffolds with optimized geometry and improved cell adhesion properties have good potential for clinical applications.

## CRediT authorship contribution statement

**Yakui Liu:** Writing – review & editing, Writing – original draft, Visualization, Software, Resources, Methodology, Investigation, Formal analysis, Data curation, Conceptualization. **Max von Witzleben:** Writing – review & editing, Supervision, Methodology, Investigation. **Sarah Duin:** Writing – review & editing, Supervision, Software. **Anne Bernhardt:** Writing – review & editing, Supervision, Software, Data curation. **Michael Gelinsky:** Writing – review & editing, Supervision, Resources, Project administration, Funding acquisition.

## Ethics approval and consent to participate

Fresh abdominal wall tissues from cadaver pigs ( provided by Pulmonary Engineering Group, Department of Anesthesiology and Intensive Care Medicine, University Hospital Carl Gustav Carus) were collected and frozen within 2 h post-harvest. The secondary use of this cadaveric porcine tissue for abdominal wall patch testing did not require additional ethical approval, as confirmed by the institutional Animal Ethics Committee, since the tissue originated from a pre-approved animal study.

## Funding

Yakui Liu acknowledges funding support from the 10.13039/501100004543China Scholarship Council (Grant No. 202206890038).

## Declaration of competing interest

The authors declare that they have no known competing financial interests or personal relationships that could have appeared to influence the work reported in this paper.
