# Supplementary material for: Topology-optimized melt-electrowritten PCL patch for abdominal wall reconstruction
Source: Bioact Mater. 2025 Oct 1;55:529–45. doi: 10.1016/j.bioactmat.2025.09.026 (PMC12522717; doi:10.1016/j.bioactmat.2025.09.026)
Supplement: Multimedia component 1 [file mmc1.doc]

**Figure S1.** **Topology optimization setup**. (A) Model geometry used for the topology optimization, depicting a 10 mm x 10 mm x 0.1 mm PCL sheet. (B) Schematic of the loading and boundary conditions, where the external forces (16 N) are applied at two sides of the scaffold, with the remaining two sides fixed. The goal of optimization is to reduce weight. (C) Three different control conditions. (D) Re-evaluation of the optimized geometry.


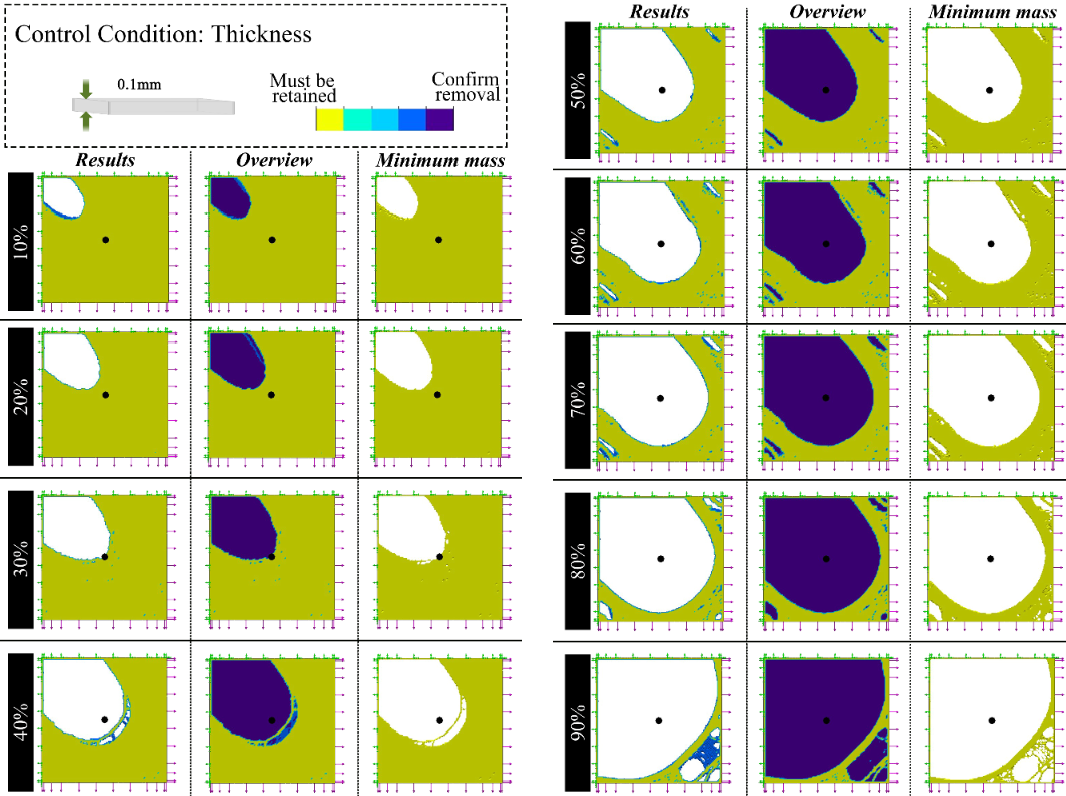


**Figure S2**. Topology optimization results for the thickness control condition. Each row represents a different level of material removal from 10% to 90%. The color gradient indicates retained areas in yellow and regions confirmed for removal in different shades of blue.


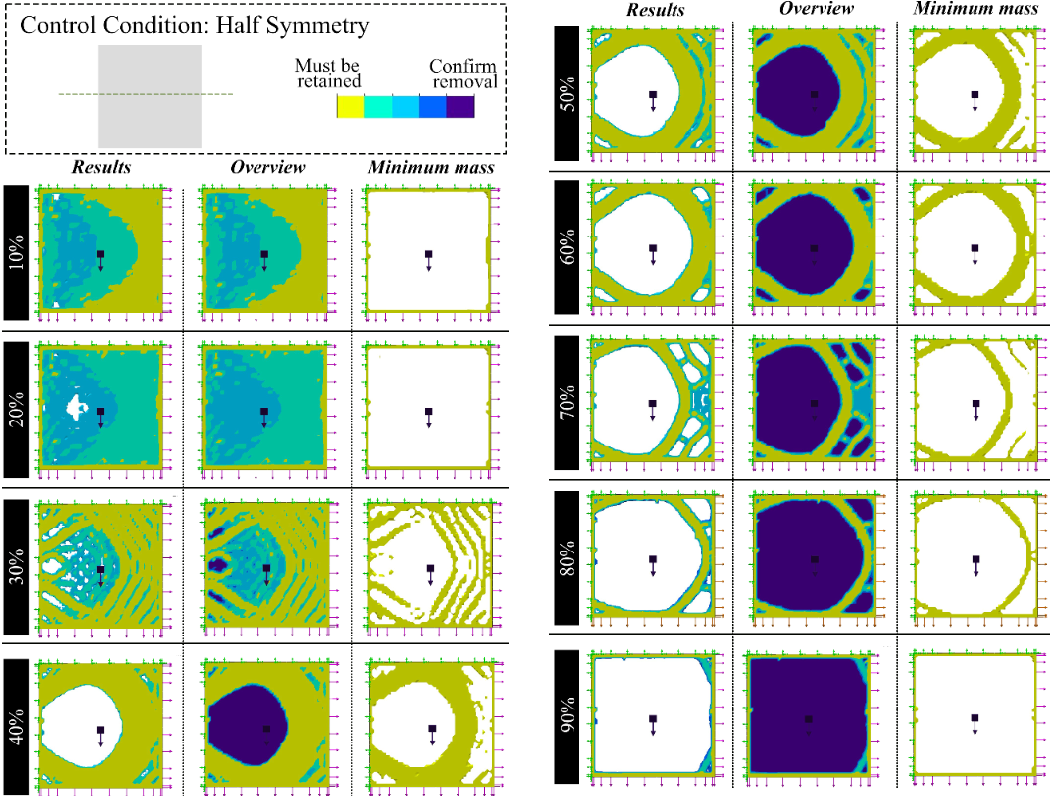


**Figure S3**. Topology optimization results for the half symmetry control condition. Each row represents a different level of material removal from 10% to 90%. The color gradient indicates retained areas in yellow and regions confirmed for removal in different shades of blue. It is important to note that the simulation for the 90% material removal condition could not be completed due to computational limitations, which led to system crashes during processing.


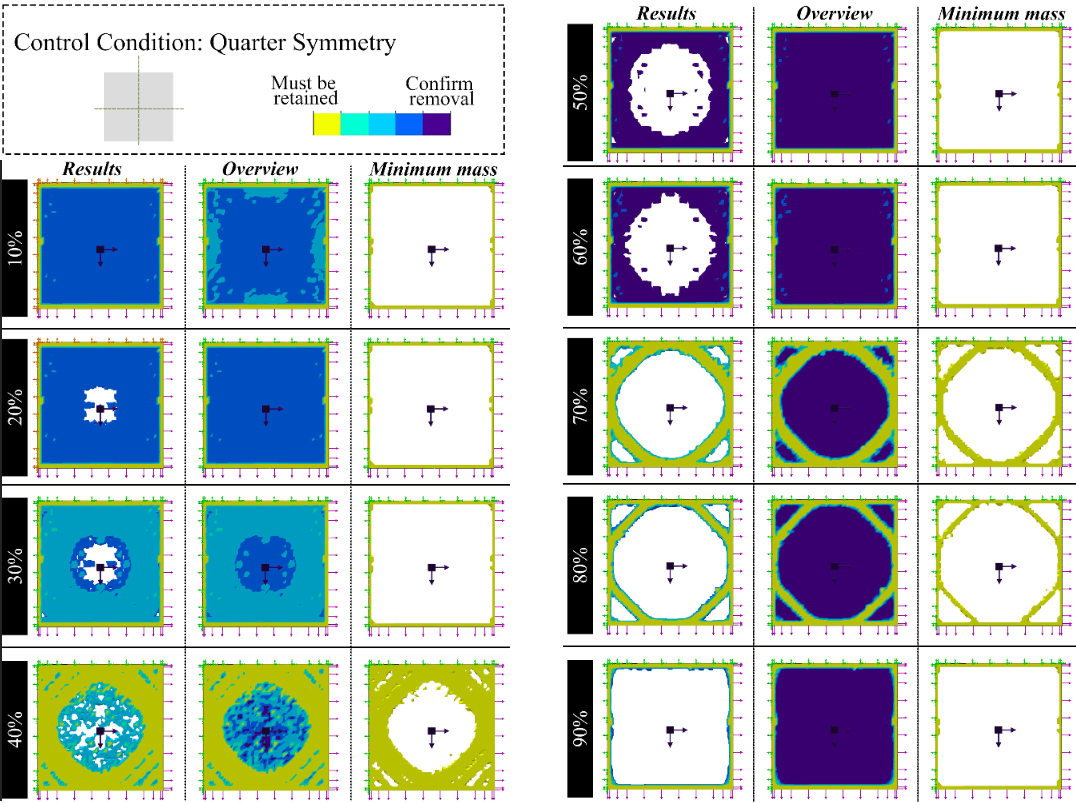


**Figure S4**. Topology optimization results for the quarter symmetry control condition. Each row represents a different level of material removal from 10% to 90%. The color gradient indicates retained areas in yellow and regions confirmed for removal in different shades of blue. It is important to note that the simulation for the 90% material removal condition could not be completed due to computational limitations, which led to system crashes during processing.

**
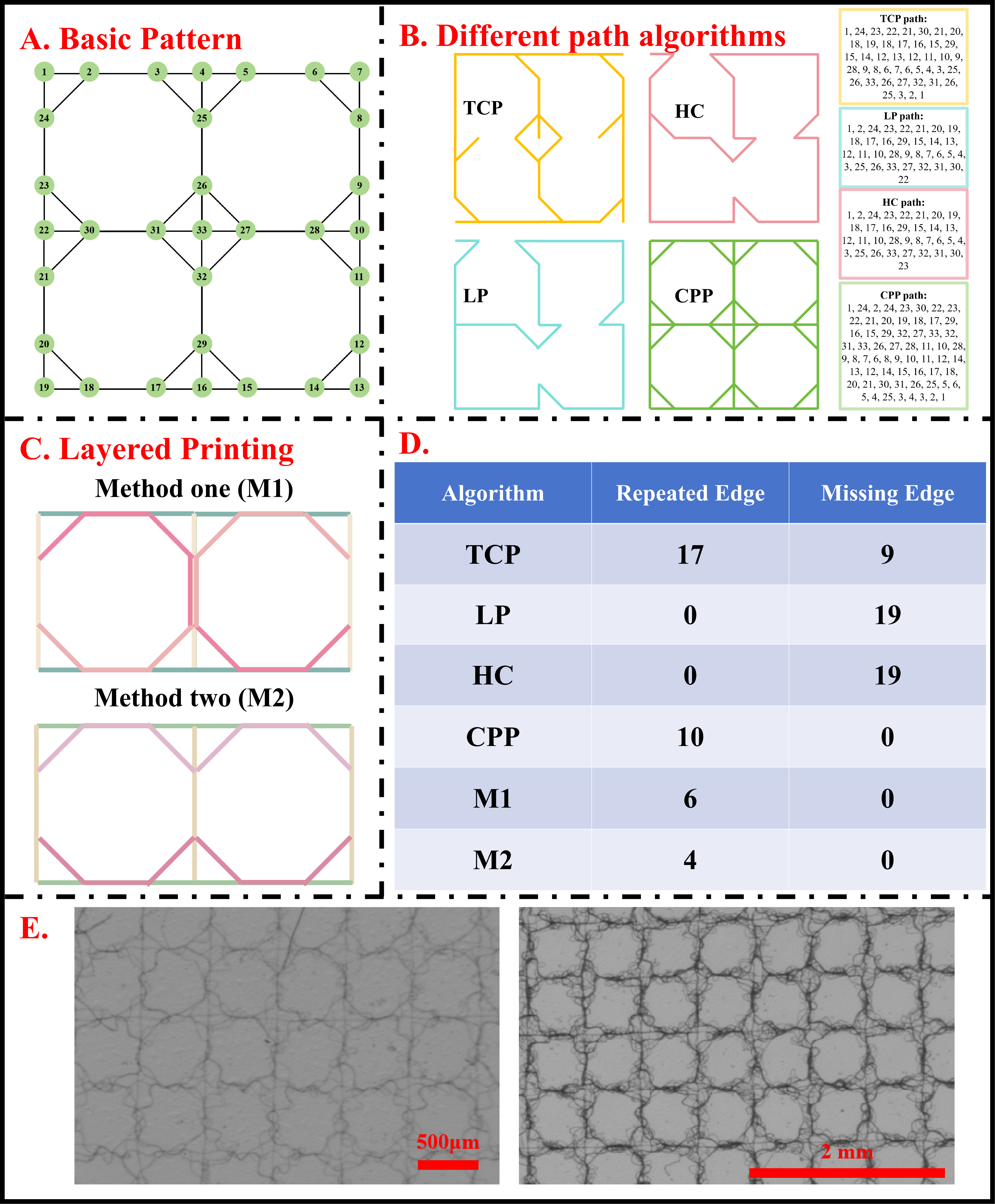
**

**Figure S5**. (A) Basic pattern. (B) Different path planning algorithms used for printing, including TCP (Traveler's Problem Path), LP (Longest Path), HC (Hamiltonian Path), and CPP (Chinese Postman Path). Each algorithm is shown with its respective node sequences used to optimize the printing path. (C) Two layered printing methods (M1 and M2) show different approaches to layer stacking. (D) Summary table comparing the effectiveness of each algorithm regarding repeated and missing edges. (E) Scaffolds printed using the M1 and M2 printing paths.


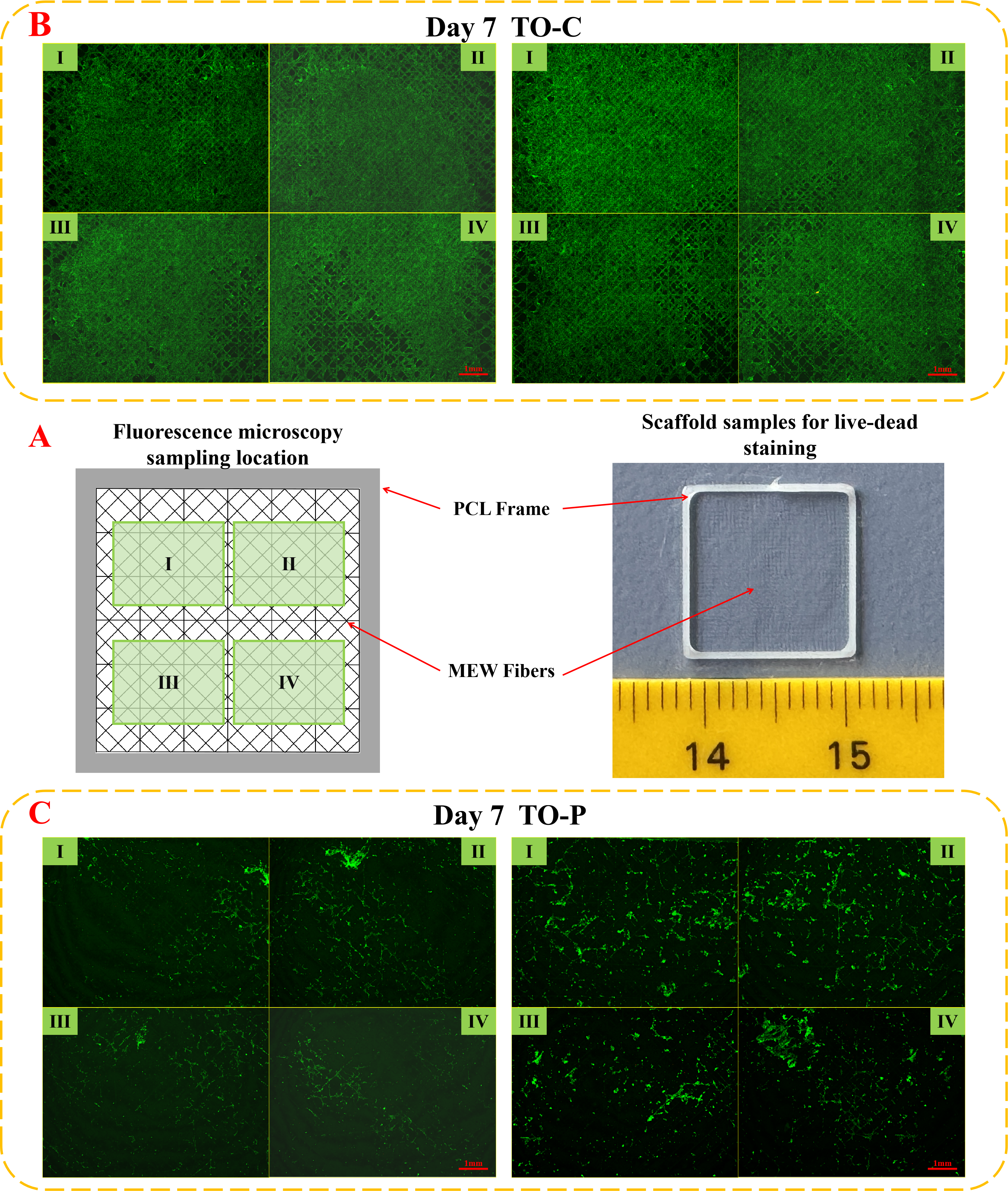


**Figure S6**. Assessment of NHDF uniformity on thin (4 layers) scaffolds at day 7. (A) Sampling strategy: a 10 × 10 mm scaffold with a PCL frame was divided into four 2× microscope fields (I–IV, green boxes), which together cover almost the entire scaffold. (B) Live/dead images of TO-C on day 7. (C) Live/dead images of TO-P on day 7. Scale bar: 1mm.


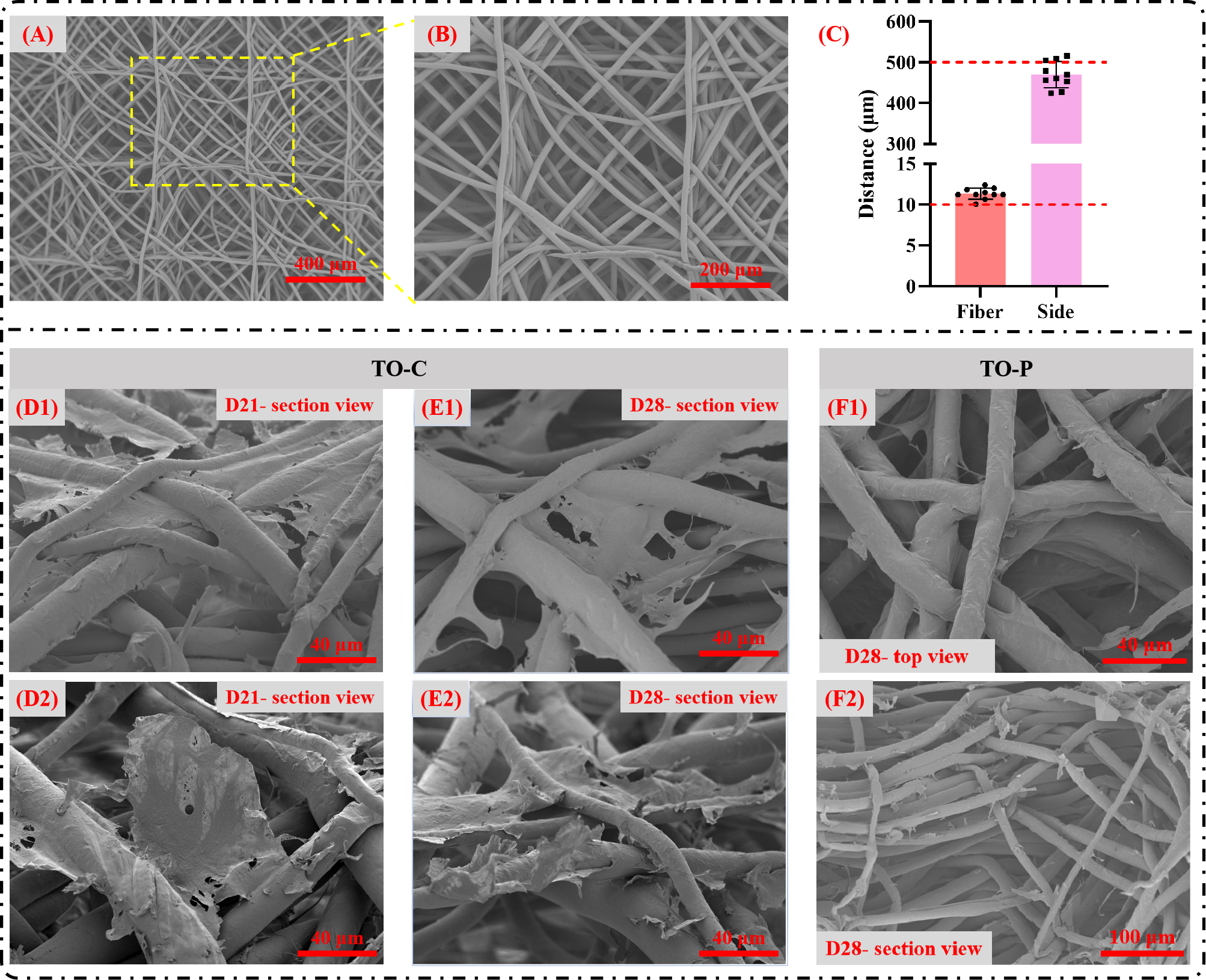


**Figure S7**. Morphology and cell infiltration of the 300-layer TO scaffold. (A) Low-magnification and (B) higher-magnification SEM overview show the structure of 300-layer TO scaffold; minor filament offsets do not disturb the global architecture. (C) Quantitative measurements (n = 10) about that fiber diameter and side length. (D1,D2) Cross-sections at day 21 and (E1,E2) day 28 reveal a continuous sheet-like cell/ECM layer interwoven with the fibers in TO-C. (F1,F2) Top view and cross-sections at day 28 in TO-P.


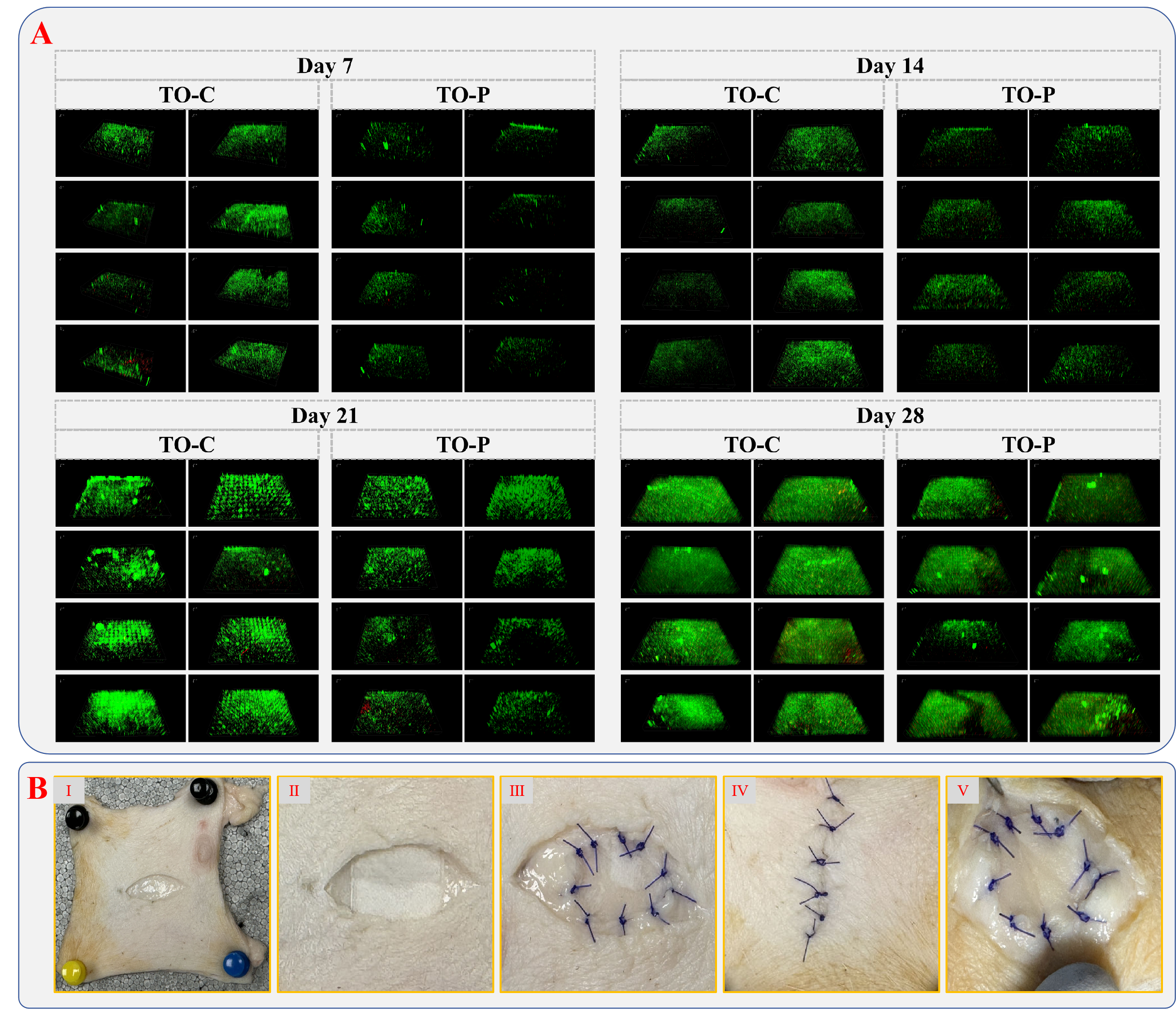


**Figure S8**. (A) Live cell staining for computational biomechanics. (B) Steps of in vitro porcine model. Ⅰ: Defect preparation; Ⅱ: implant scaffold; Ⅲ: suture scaffold; Ⅳ: suture defect; Ⅴ: after testing.

**Appendix**

**Path planning code:**

**① The relevant code of the algorithm in Figure 1B:**

import networkx as nx

import matplotlib.pyplot as plt

G = nx.Graph()

edges = [

(1, 2), (2, 3), (3, 4), (4, 5), (5, 6), (6, 7), (7, 8), (8, 9),(9, 10), (10, 11), (11, 12), (12, 1), (2, 12), (3, 5), (6, 8), (9, 11)

]

G.add_edges_from(edges)

positions = {

1: (0, 3), 2: (1, 3), 3: (2, 3), 4: (3, 3),

5: (3, 2), 6: (3, 1), 7: (3, 0), 8: (2, 0),

9: (1, 0), 10: (0, 0), 11: (0, 1), 12: (0, 2)

}

if nx.is_eulerian(G):

eulerian_path = list(nx.eulerian_circuit(G))

eulerian_edges = [(eulerian_path[i][0], eulerian_path[i][1]) for i in range(len(eulerian_path))]

print("Eulerian Path:", [edge[0] for edge in eulerian_path] + [eulerian_path[0][0]])

plt.figure(figsize=(6, 6))

nx.draw(G, pos=positions, with_labels=True, node_color='lightblue', edge_color='black', node_size=700, font_size=10)

nx.draw_networkx_edges(G, pos=positions, edgelist=eulerian_edges, edge_color='red', width=2)

plt.title("Eulerian Path")

plt.show()

else:

print("No Eulerian Path")

tsp_path = nx.approximation.traveling_salesman_problem(G)

tsp_edges = [(tsp_path[i], tsp_path[i+1]) for i in range(len(tsp_path)-1)] + [(tsp_path[-1], tsp_path[0])]

print("TSP Path:", tsp_path)

plt.figure(figsize=(6, 6))

nx.draw(G, pos=positions, with_labels=True, node_color='lightblue', edge_color='black', node_size=700, font_size=10)

nx.draw_networkx_edges(G, pos=positions, edgelist=tsp_edges, edge_color='blue', width=2)

plt.title("Traveling Salesman Problem Path (TSP)")

plt.show()

matching_edges = list(nx.max_weight_matching(G, maxcardinality=True))

print("Maximal Path Cover Edges:", matching_edges)

plt.figure(figsize=(6, 6))

nx.draw(G, pos=positions, with_labels=True, node_color='lightblue', edge_color='black', node_size=700, font_size=10)

nx.draw_networkx_edges(G, pos=positions, edgelist=matching_edges, edge_color='green', width=2)

plt.title("Maximal Path Cover (Matching Approximation)")

plt.show()

cpp_graph = nx.eulerize(G)

cpp_edges = list(cpp_graph.edges())

print("Chinese Postman Path (Edges with repetition):", cpp_edges)

plt.figure(figsize=(6, 6))

nx.draw(G, pos=positions, with_labels=True, node_color='lightblue', edge_color='black', node_size=700, font_size=10)

nx.draw_networkx_edges(G, pos=positions, edgelist=cpp_edges, edge_color='purple', width=2)

plt.title("Chinese Postman Path (CPP)")

plt.show()

try:

shortest_path = nx.shortest_path(G, source=1, target=7)

shortest_path_edges = [(shortest_path[i], shortest_path[i+1]) for i in range(len(shortest_path)-1)]

print("Shortest Path from 1 to 7:", shortest_path)

plt.figure(figsize=(6, 6))

nx.draw(G, pos=positions, with_labels=True, node_color='lightblue', edge_color='black', node_size=700, font_size=10)

nx.draw_networkx_edges(G, pos=positions, edgelist=shortest_path_edges, edge_color='orange', width=2)

plt.title("Shortest Path from 1 to 7")

plt.show()

except nx.NetworkXNoPath:

print("No path from 1 to 7")

②**The relevant code of the algorithm in Figure S5B:**

import networkx as nx

import matplotlib.pyplot as plt

G = nx.Graph()

edges = [

(1, 2), (2, 3), (3, 4), (4, 5), (5, 6), (6, 7), (7, 8), (8, 9),

(9, 10), (10, 11), (11, 12), (12, 13), (13, 14), (14, 15), (15, 16),

(16, 17), (17, 18), (18, 19), (19, 20), (20, 21), (21, 22), (22, 23),

(23, 24), (24, 1), (2, 24), (3, 25), (4, 25), (5, 25), (6, 8), (25, 26),

(23, 30), (22, 30), (21, 30), (30, 31), (31, 33), (31, 26), (31, 32),

(26, 33), (33, 32), (26, 27), (32, 27), (33, 27), (27, 28), (28, 9),

(28, 10), (28, 11), (20, 18), (32, 29), (29, 17), (29, 16), (29, 15),

(14, 12)

]

G.add_edges_from(edges)

positions = {

1: (0, 6), 2: (1, 6), 3: (2, 6), 4: (3, 6), 5: (4, 6), 6: (5, 6),

7: (6, 6), 8: (6, 5), 9: (6, 4), 10: (6, 3), 11: (6, 2), 12: (6, 1),

13: (6, 0), 14: (5, 0), 15: (4, 0), 16: (3, 0), 17: (2, 0), 18: (1, 0),

19: (0, 0), 20: (0, 1), 21: (0, 2), 22: (0, 3), 23: (0, 4), 24: (0, 5),

25: (3, 5), 26: (3, 4), 27: (4, 3), 28: (5, 3), 29: (3, 1),

30: (1, 3), 31: (2, 3), 32: (3, 2), 33: (3, 3)

}

if nx.is_eulerian(G):

eulerian_path = list(nx.eulerian_circuit(G))

eulerian_edges = [(eulerian_path[i][0], eulerian_path[i][1]) for i in range(len(eulerian_path))]

print("Eulerian Path:", [edge[0] for edge in eulerian_path] + [eulerian_path[0][0]])

plt.figure(figsize=(10, 10))

nx.draw(G, pos=positions, with_labels=True, node_color='lightblue', edge_color='black', node_size=500, font_size=8)

nx.draw_networkx_edges(G, pos=positions, edgelist=eulerian_edges, edge_color='red', width=2)

plt.title("Eulerian Path")

plt.show()

else:

print("No Eulerian Path")

tsp_path = nx.approximation.traveling_salesman_problem(G)

tsp_edges = [(tsp_path[i], tsp_path[i+1]) for i in range(len(tsp_path)-1)] + [(tsp_path[-1], tsp_path[0])]

print("TSP Path:", tsp_path)

plt.figure(figsize=(10, 10))

nx.draw(G, pos=positions, with_labels=True, node_color='lightblue', edge_color='black', node_size=500, font_size=8)

nx.draw_networkx_edges(G, pos=positions, edgelist=tsp_edges, edge_color='blue', width=2)

plt.title("Traveling Salesman Problem Path (TSP)")

plt.show()

matching_edges = list(nx.max_weight_matching(G, maxcardinality=True))

print("Maximal Path Cover Edges:", matching_edges)

plt.figure(figsize=(10, 10))

nx.draw(G, pos=positions, with_labels=True, node_color='lightblue', edge_color='black', node_size=500, font_size=8)

nx.draw_networkx_edges(G, pos=positions, edgelist=matching_edges, edge_color='green', width=2)

plt.title("Maximal Path Cover (Matching Approximation)")

plt.show()

cpp_graph = nx.eulerize(G)

cpp_edges = list(cpp_graph.edges())

cpp_path = list(nx.eulerian_circuit(cpp_graph))

cpp_path_nodes = [edge[0] for edge in cpp_path] + [cpp_path[0][0]]

print("Chinese Postman Path (Node Order):", cpp_path_nodes)

plt.figure(figsize=(10, 10))

nx.draw(G, pos=positions, with_labels=True, node_color='lightblue', edge_color='black', node_size=500, font_size=8)

cpp_edges_list = [(cpp_path[i][0], cpp_path[i][1]) for i in range(len(cpp_path))]

nx.draw_networkx_edges(G, pos=positions, edgelist=cpp_edges_list, edge_color='purple', width=2)

plt.title("Chinese Postman Path (CPP)")

plt.show()

def dfs_longest_path(graph, node, visited, path):

visited.add(node)

path.append(node)

longest_path = list(path)

for neighbor in graph.neighbors(node):

if neighbor not in visited:

candidate_path = dfs_longest_path(graph, neighbor, visited, path)

if len(candidate_path) > len(longest_path):

longest_path = candidate_path

path.pop()

visited.remove(node)

return longest_path

all_longest_paths = []

for start_node in G.nodes:

visited = set()

path = []

longest_path_from_node = dfs_longest_path(G, start_node, visited, path)

all_longest_paths.append(longest_path_from_node)

longest_path = max(all_longest_paths, key=len)

longest_path_edges = [(longest_path[i], longest_path[i+1]) for i in range(len(longest_path)-1)]

print("Longest Path (No Repeated Edges):", longest_path)

plt.figure(figsize=(10, 10))

nx.draw(G, pos=positions, with_labels=True, node_color='lightblue', edge_color='black', node_size=500, font_size=8)

nx.draw_networkx_edges(G, pos=positions, edgelist=longest_path_edges, edge_color='orange', width=2)

plt.title("Longest Path without Repeated Edges")

plt.show()
